# p53 Gene Repair with Zinc Finger Nucleases Optimised by Yeast 1-Hybrid and Validated by Solexa Sequencing

**DOI:** 10.1371/journal.pone.0020913

**Published:** 2011-06-09

**Authors:** Frank Herrmann, Mireia Garriga-Canut, Rebecca Baumstark, Emmanuel Fajardo-Sanchez, James Cotterell, André Minoche, Heinz Himmelbauer, Mark Isalan

**Affiliations:** 1 EMBL/CRG Systems Biology Research Unit, Centre for Genomic Regulation (CRG) and UPF, Barcelona, Spain; 2 Max Planck Institute for Molecular Genetics, Berlin, Germany; 3 Ultrasequencing Unit, Centre for Genomic Regulation and UPF, Barcelona, Spain; University of Medicine and Dentistry of New Jersey, United States of America

## Abstract

The tumor suppressor gene p53 is mutated or deleted in over 50% of human tumors. As functional p53 plays a pivotal role in protecting against cancer development, several strategies for restoring wild-type (wt) p53 function have been investigated. In this study, we applied an approach using gene repair with zinc finger nucleases (ZFNs). We adapted a commercially-available yeast one-hybrid (Y1H) selection kit to allow rapid building and optimization of 4-finger constructs from randomized PCR libraries. We thus generated novel functional zinc finger nucleases against two DNA sites in the human p53 gene, near cancer mutation ‘hotspots’. The ZFNs were first validated using *in vitro* cleavage assays and *in vivo* episomal gene repair assays in HEK293T cells. Subsequently, the ZFNs were used to restore wt-p53 status in the SF268 human cancer cell line, via ZFN-induced homologous recombination. The frequency of gene repair and mutation by non-homologous end-joining was then ascertained in several cancer cell lines, using a deep sequencing strategy. Our Y1H system facilitates the generation and optimisation of novel, sequence-specific four- to six-finger peptides, and the p53-specific ZFN described here can be used to mutate or repair p53 in genomic loci.

## Introduction

The tumor suppressor p53 serves as a “guardian of the genome” [1] and has been studied intensively for over 30 years. By responding to cellular stresses, such as DNA damage, hypoxia and cell-cycle aberrations, p53 is activated as a transcription factor. p53 can thus help to promote the repair and survival of damaged cells by inducing cell-cycle arrest, or it can promote the permanent removal of damaged cells by inducing programmed cell death or senescence [2].

As the p53 gene is either mutated or deleted in more than 50% of human tumors [3], functional p53 is very important in protecting against cancer development. The vast majority of p53 mutations in human tumors are single missense mutations that cluster in the core DNA-binding domain of the protein (residues 100–300). This leads to both the disruption of normal p53 function and the accumulation of high levels of mutant p53 with various gain-of-function activities (reviewed in [4], [5]). Since tumors with mutant p53 often present increased chemo- and radio-resistance, mutant p53 is an appealing molecular target for tumor suppression. Moreover, the restoration of p53 function is considered an important issue in cancer therapy [6], [7], [8]. Several therapeutic strategies have therefore been pursued, including expressing wt-p53 in gene therapy, eliminating mutant p53 cancer cells with adenovirus, and directly restoring normal function with small molecules that alter mutant p53 conformation (reviewed in [9]).

Despite p53 being a desirable target, we currently lack the necessary tools to carry out the most direct approach: to modify genomes at will at disease loci like p53. Therefore we set out to develop just such a strategy, using a recently-developed technology: zinc finger nucleases (ZFNs). Since the first seminal publications about ZFN fusions in the late 1990s [10], [11], [12], these artificial proteins have promised to deliver a wide range of genome engineering tools (reviewed in [13], [14]). The beauty of this approach is that zinc fingers are easily re-engineered to bind a wide variety of DNA sequences (reviewed in Ref. [15]). Thus, ZFNs effectively allow a type of ‘genome sculpting’ where externally provided DNA can be recombined precisely into a genome [16], resulting in site-specific gene repair, mutation, insertion or deletion.

ZFN gene targeting was first illustrated in the case of a mutant Drosophila *yellow* gene [17], [18] and has since resulted in a whole field of engineering ZFNs. Examples include targeting disease loci, such as IL2RG (mutated in severe combined immunodeficiency; SCID-X1), where first gene repair [19], and then exogenous gene integration [20] were achieved. ZFNs have also targeted genes in model organisms such as *C. elegans* and *Drosophila* [21], [22], [23], zebrafish [24], [25], [26], mouse [27], and plants [28], [29], [30], [31], [32]. The technology has been extended to mammalian systems such as stem cells [33], [34], [35], the induction of cellular HIV resistance [36], [37], [38] and even whole rat knockouts [39], [40].

The specificity of ZFNs depends on artificially-engineered DNA-binding domains: multi-zinc finger arrays that recognise long DNA sequences. There exists a large body of work on zinc finger engineering (reviewed in [15]). Briefly, the established engineering methods (amongst others) range from rational design [41], modular assembly with pre-made fingers [42], [43], [44], overlapping finger assembly [45], [46] and bacterial-two hybrid [47].

A recent development is the emergence of two publically-available sources of zinc fingers, the academic Zinc Finger Consortium (ZFC; www.zincfingers.org) and the commercially-available CompoZr, offered by Sigma Aldrich [48]. In particular, the ZFC has provided a variety of open source tools for the community to employ [49], [50], [51], [52].

Although both sources facilitate obtaining ZFNs, these have to be tested on a case-by-case basis for in vivo functionality, and suboptimal candidates often have to be abandoned because of a lack of straightforward optimisation protocols. Whereas screening systems exist for 1- to 2-finger libraries (e.g. phage display) and 3-finger mini-libraries of pre-selected modules (B2H [47]), no straightforward system exists to optimize the 4- to 6-finger type scaffolds which are provided by Sigma.

**Figure 1:**
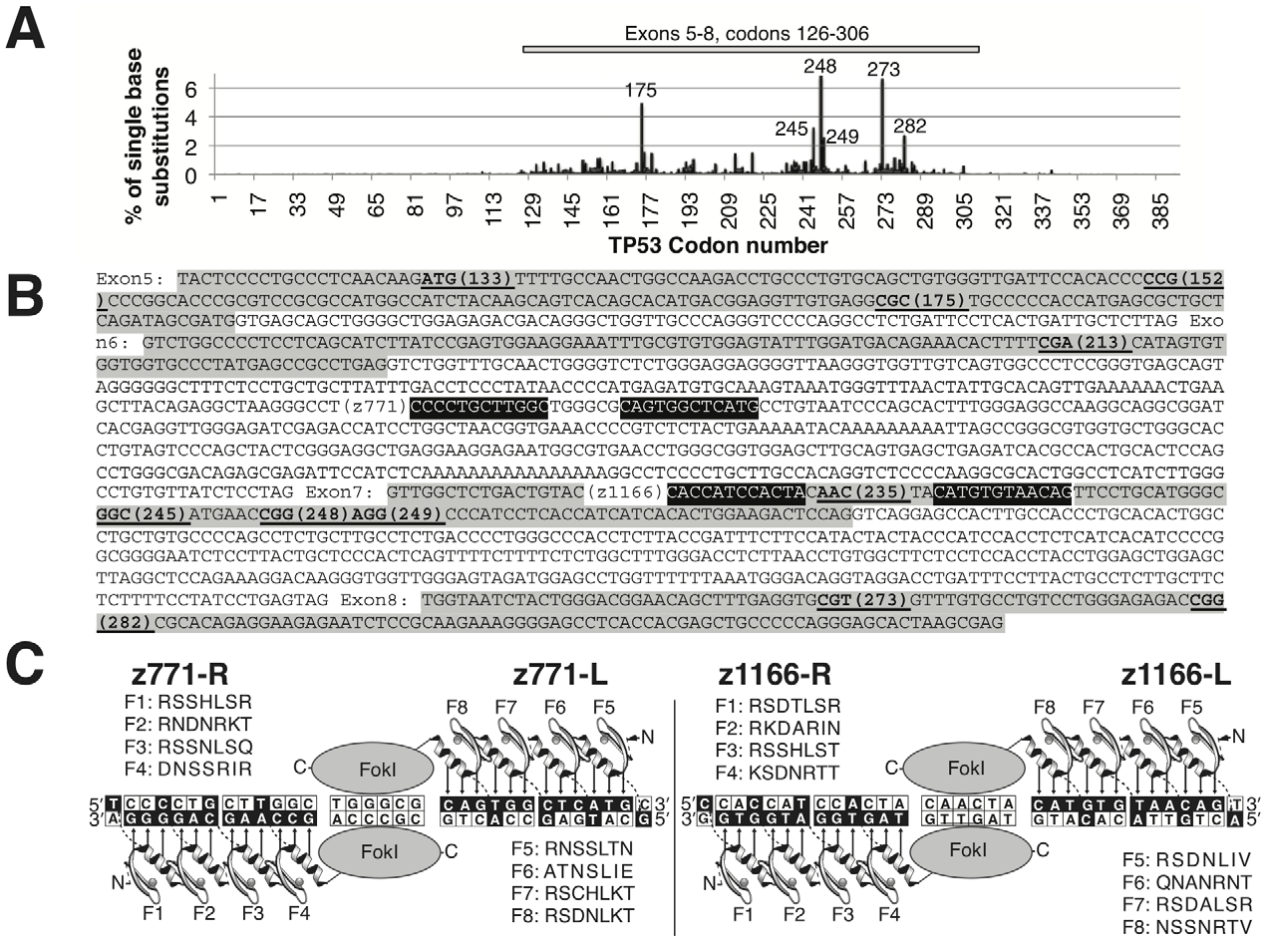
Zinc finger nucleases for the human p53 gene. (*A*) Mutation hotspots in somatic cancers from the IARC TP53 Mutation Database R13. (*B*) Exons 5–8 from the p53 gene (Human Genome: NW_001838403) are highlighted in grey and contain nearly all mutation hotspots (underlined black; codon number in brackets). ZFN binding sites are highlighted in black with white letters. (*C*) Canonical model of designed zinc finger nucleases (z771 and z1166) against two target sites in the p53 gene. Arrows indicate possible base contacts. Zinc finger alpha helix sequences, involved in DNA recognition, are indicated (F1, Finger 1, etc.).

In this study, we set out to target the p53 gene using paired 4-finger ZFN [19], and to develop a platform for optimising poly-finger constructs. Because we found that classical phage display could not handle >3-fingers (4-finger fd phage display always resulted in truncations during selection; data not shown), we modified a commercially-available yeast one-hybrid kit to optimize such libraries . This was used to generate functional ZFNs against two different sites (one exonic, one intronic) located within the human p53 gene, in close proximity to the mutation hotspots of p53 in somatic cancers (**Fig. 1**).

## Results

### Yeast one-hybrid selection of zinc fingers against two loci in the human p53 gene

**Figure 2:**
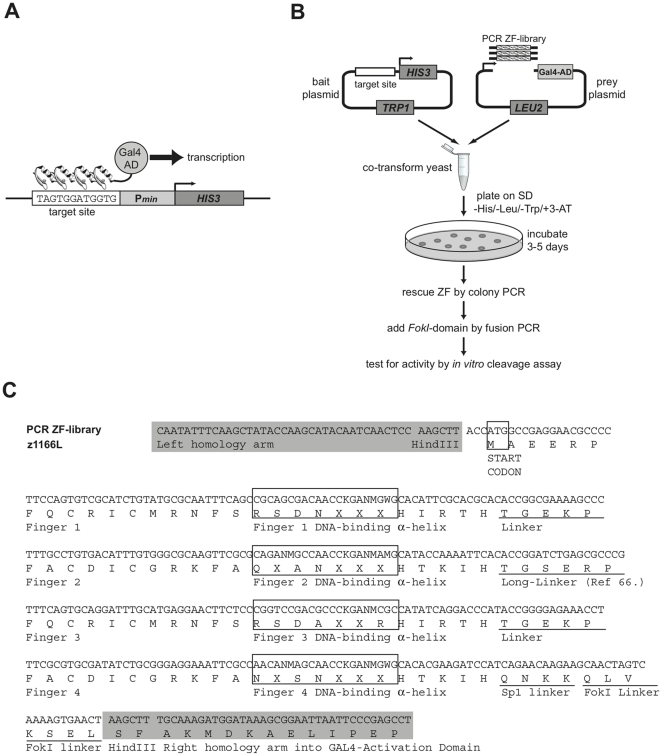
Yeast one-hybrid based selection for ZFNs. (*A*) The ZF-target site is cloned upstream of a minimal promoter (P*_min_*) and the HIS3 reporter in the bait plasmid. Any interaction between a ZFP:Gal4-AD fusion protein and the target sequence stimulates transcription of HIS3 allowing selection on His-selective medium (*B*) The ZF-library:Gal4-AD fusion is generated by yeast recombination of a PCR-generated four-finger library cassette with the linearized prey plasmid; no extra library cloning step is required. Thus, bait plasmid, linearized prey plasmid and library PCR cassette are co-transformed into yeast. After incubation for 3–5 days, expression from the HIS3 reporter is detected in colonies that are able to grow on a selection medium that lacks histidine and contains 3-AT (see Methods). ZFP from positive clones are rescued by colony-PCR, are fused to a *FokI*-domain and are tested for activity by an *in vitro* cleavage assay. (*C*) Zinc finger library PCR template (z1166L). The template is based on 2×2-finger units from F2-F3 of the Zif268 sequence [65]. Each pair of 2-finger units is separated by a longer TGSERP linker [66]. The final linker, (QNKKQLVKSEL) is compatible with the FokI sequence and is adapted from [16]. DNA-recognition helices are selectively randomised at certain positions (marked “X”). Full sequences and randomisation strategy are in **Methods S1**.

One-hybrid screening in yeast is a powerful method to rapidly identify DNA-binding peptides that can interact with a specific DNA sequence of interest. We therefore developed a yeast one-hybrid (Y1H) selection system for zinc finger peptides, based on the commercially-available Matchmaker Kit (Clontech). The system allowed us to construct semi-randomised zinc finger libraries by PCR (without cloning), and to screen them in one step, by yeast transformation and plating on selective medium (**Fig. 2**).

We constructed four zinc finger libraries against two sites in the human p53 gene (with two sub-sites L/R each for a functional ZFN configuration; see **Fig. 1B,C**). The libraries were rationally designed, based on the established protein-DNA recognition code [53] and previously successful library designs [46]. Thus, the libraries were randomized at several base-contacting positions in the alpha-helix domains of the zinc fingers (library designs and randomisation strategy are listed in **Methods S1**). The four-finger library cassettes were built from degenerate oligonucleotides, via a PCR-based construction [54], and were transformed directly into yeast, together with linearized “prey plasmid” (pGADT7-Rec2) and the “bait plasmids” with the target DNA sequences (**Fig. 2B**). The system exploits the high rate of recombination in yeast to bypass standard cloning and to allow the PCR library to fuse in frame with the prey vector, in the single selection step.

A further modification to the Y1H, that we found to be essential for selecting ZFP, was that the library cassettes had to be introduced N-terminal to the Gal4-AD, rather than C-terminal as in the Matchmaker kit (Clontech)(An example of a PCR cassette, complete with homology arms, ready for transformation into yeast is provided in **Methods S1**). The new configuration reduces the number of false positive clones arising from truncated peptides binding to the target sites; N-terminal truncations result in frameshifts or deletion of the Gal4-AD, in most cases.

To create the “bait plasmids”, the target DNA sequences for the four ZFNs z771L, z771R, z1166L and z1166R) were cloned in single copies, upstream of a minimal promoter controlling the *HIS3* reporter gene (**Fig. 1C** **and** **2**). Successful application of any Y1H system is constrained by requiring low recognition of the target sequence by endogenous transcription factors. However, the basal expression of the His3 protein, in the absence of an activating prey protein, can be repressed by using 3-AT (a competitive inhibitor of the His3 enzyme) [55]. Thus, in the presence of increasing amounts of 3-AT, more His3 needs to be expressed to confer growth and so the selection pressure can be fine-tuned. Before screening the libraries, we tested each target site for basal histidine expression in the absence of activating zinc finger proteins. The amount of 3-AT needed to fully suppress basal expression varied between 25mM (z1166L) and 75mM (z771L, z771R, z1166R). Consequently, 100–150 mM 3-AT was used for screening the libraries for DNA-binding ZFP. It is worth noting that, classically, Y1H uses chromosomal integration of the bait plasmid to reduce basal expression. However, the Clontech kit uses low-copy-number plasmids to obviate this need (Clontech Protocol No. PT3529-1).

To interrogate the zinc finger libraries in our Y1H assay, they were co-transformed as PCR products, together with linearized prey plasmids and bait plasmids, into the yeast strain Y187, as described in the manual of the Matchmaker Kit (Clontech). Transformations were plated on selection media containing 3-AT and lacking histidine, tryptophan and leucine. The number of screened clones per library was calculated according to the manual of the Matchmaker Kit (Clontech) and was typically 100 000–160 000 for each experiment. After incubation for 3–5 days at 30°C, library screening revealed many potentially positive colonies of various sizes (200 to 2000 colonies). Ultimately, 96 colonies were picked for each library and were replated in a 96-well pattern, on selection media containing 3-AT.

**Figure 3:**
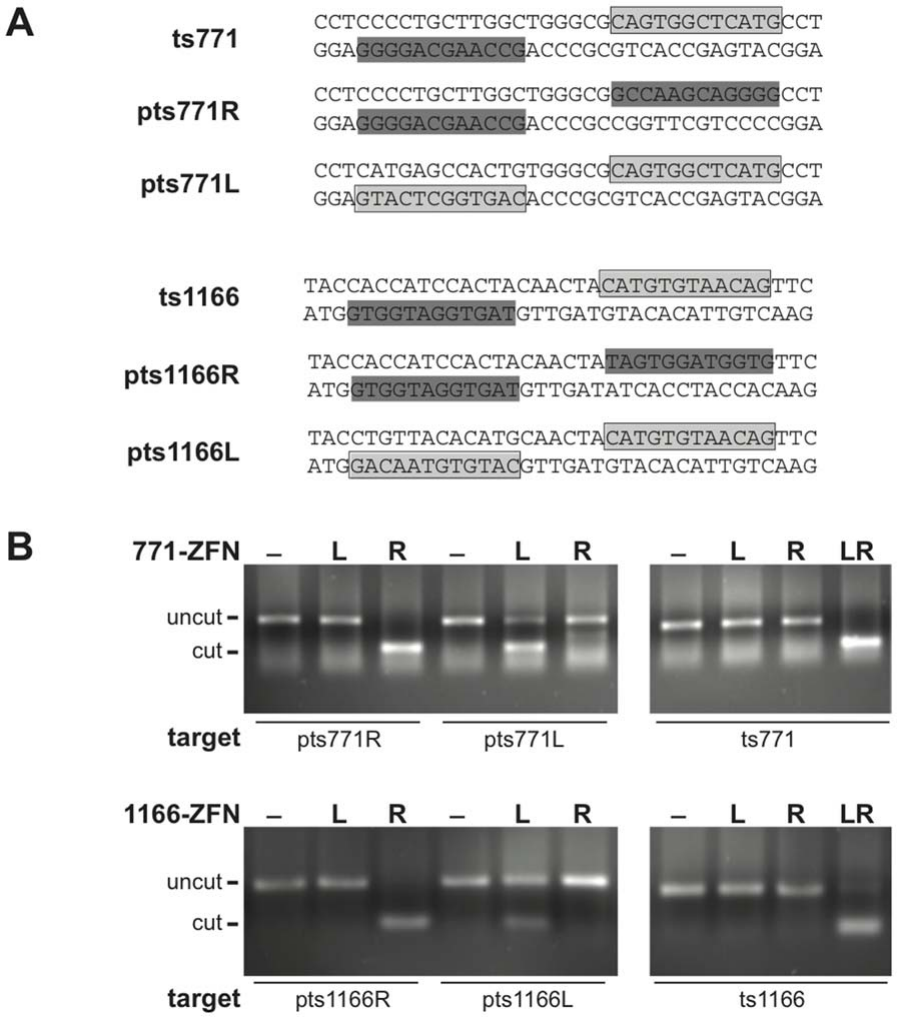
*In vitro* cleavage of p53 zinc finger nucleases. (*A*) Schematic of palindromic- (pts) or heterodimer (ts) DNA target sites of z771 and z1166 ZFNs. Both strands of DNA are shown with the top strand written 5′ to 3′ and the bottom strand written 3′ to 5′. The primary strand of the 12-bp target sites is highlighted (ZFNs ‘771L’ and ‘1166L’ in light grey, ‘771R’ and ‘1166R’ in dark grey). (*B*) Analysis of homo- and heterodimer cleavage reactions. In vitro expressed ZFNs were incubated with a linear target DNA substrate and cleavage products were analyzed by agarose gel electrophoresis. Cleavage of the target DNA results in two DNA molecules of the same size, simplifying the analysis.

Although this workflow undersampled both the theoretical library sizes (**Methods S1**) and potential positives, it was sufficient to obtain potentially-functional ZFP in one transformation step, starting from rational designs that had little or no activity in DNA cleavage assays, such as those shown in Fig. 3. The next step was therefore to test these candidates for ZFN cleavage activity.

### In vitro cleavage of p53 target sites

To validate Y1H-based protein-DNA interactions, ZFP genes were recovered from yeast colonies by PCR, while introducing a T7 promoter for subsequent expression. After a PCR-based fusion to the *FokI* domain, the full length ZFN candidates were expressed *in vitro* and tested for specific cutting activity in an *in vitro* cleavage assay (see Methods and **Fig. S1**). This approach allowed us to identify several 4-finger anti-p53 ZFNs that bind and cleave their palindromic target sites efficiently *in vitro* (z771L, 4 clones; z771R, 1 clone; z1166R, 3 clones; z1166L, 1 clone).

From these clones, the best ZFN were similarly tested in homo- and heterodimer pairs, for cleaving either palindromic test sites or full DNA target sites (in the configuration shown in **Fig. 1C**; L = left; R = right). The L/L and R/R-homodimers only cut their respective palindromic target sites but not the unspecific targets (**Fig. 3B**; left panel). Conversely, the full heterodimer targets (ts771 and ts1166) were cleaved only by the correct combination of z771L/R or z1166L/R, respectively, showing that a full pair of ZFN are required for cutting (**Fig. 3B**; right panel).

### Episomal gene repair

**Figure 4:**
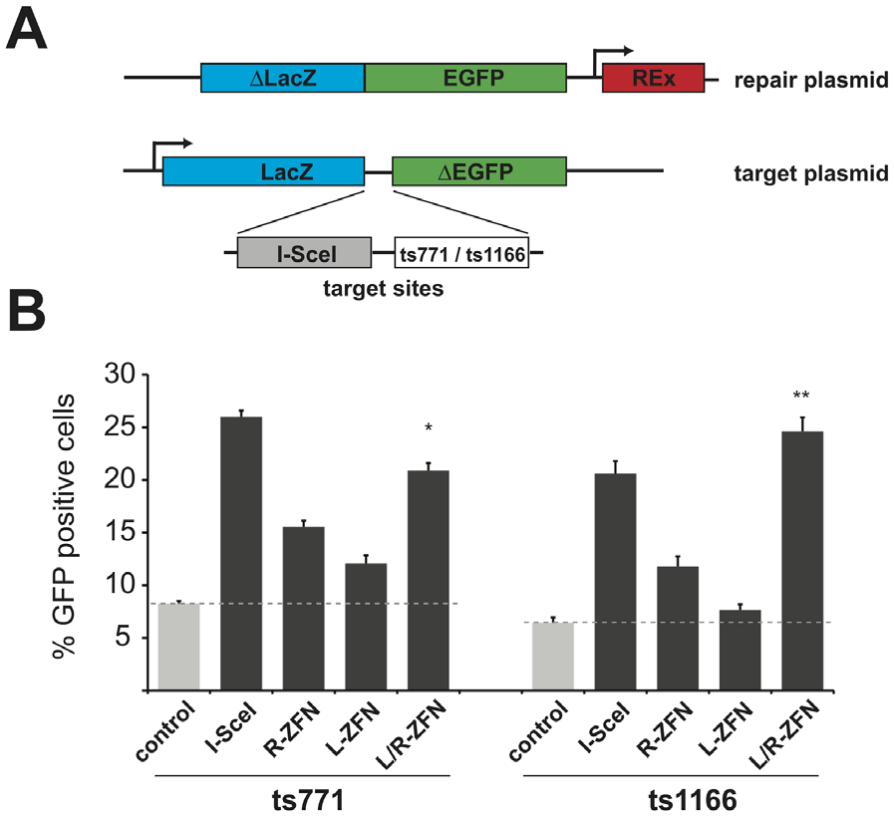
Episomal gene repair assay in HEK293T cells. (*A*) Schematic representation of the experimental setup. (*B*) Cells transfected with repair plasmid, target plasmid and ZFN expression vectors were analyzed after 48 hrs by flow cytometry. The diagram displays the fraction of EGFP-positive cells normalized for transfection efficiency. Statistically significant increases in homologous recombination (HR), compared to non-induced HR control (dashed grey line) are indicated with asterisks (** = p<0.0001 and * = p<0.002).

To evaluate whether our potential *p53*-specific ZFNs (z771 and z1166) could promote homologous recombination or gene repair *in vivo*, they were first tested in a plasmid-based EGFP repair assay, developed by the Cathomen lab [56] (**Fig. 4A**).

In this assay, a promoterless EGFP sequence, with lacZ gene homology arm, is used to repair a 5′-truncated (non-fluorescent) EGFP gene, and the process is stimulated by cleavage with the appropriate nuclease. The target plasmid harbors a 18-bp recognition binding site for the meganuclease I-*Sce*I, which serves as a positive control, in combination with a target site for the anti-p53 ZFNs, z771 or z1166. The system is thus designed to restore EGFP expression by generating a lacZ-EGFP fusion protein upon nuclease-induced homologous recombination. The expression of the red-fluorescent protein DsRed-Express (REx), from a gene cassette located on the repair plasmid, labels transfected cells [56].

To validate the efficiency of our ZFNs, we transfected HEK293T cells with either target plasmid “ts771” or “ts1166”, the repair plasmid and the respective PGK-driven ZFN pairs z771L/R and z1166L/R. 48 hours after transfection, the percentage of green and red cells was assessed by flow cytometry (**Fig. 4B**). The GFP repair assay revealed that the zinc finger nucleases showed the strongest activity when expressed in appropriate pairs to form heterodimers. The repair efficiencies approached that of the benchmark control, the meganuclease I-SceI (z771L/R, 20.9%; z1166L/R, 24.5%; I-*Sce*I, 20%–25%).

Some gene repair was observed when z771L (12%) and z771R (15%) were expressed alone, probably due to DNA-binding by one ZFN monomer, followed by non-specific FokI dimerisation. This was only seen for the nucleases with stronger activity (e.g. z1166L alone did not considerably activate HR). Recent advances in generating obligate heterodimer ZFN have demonstrated that it is possible to remove this activity [57], [58], and we used such mutants in downstream assays. Notably, the EGFP-background level in the absence of a nuclease was relatively high, representing spontaneous homologous recombination events in this episomal system (8.2% for target plasmid ts771 and 6.4% for ts1166). Nonetheless, the nuclease-induced signals were highly reproducible and statistically significant (z771L/R, p<0.002; z1166L/R, p<0.001). Therefore this assay is a good way of validating ZFN for cellular use.

### Chromosomal targeting of p53 gene by ZFNs

To determine whether our custom built ZFNs would also work on a genomic level, we transfected HEK293T cells with PGK-driven ZFN expression vectors against the target sites z771 and z1166, together with homology repair plasmids. Because p53 cancer mutations are localised to one region of hotspots (**Fig. 1A**), repair plasmids covering the majority of hotspots could be synthesised, containing either 1.35 kb or 1.78 kb DNA, homologous to the genomic *p53* locus between exons 5–8 or 6–8 (Sequences given in **Methods S1**).

**Figure 5:**
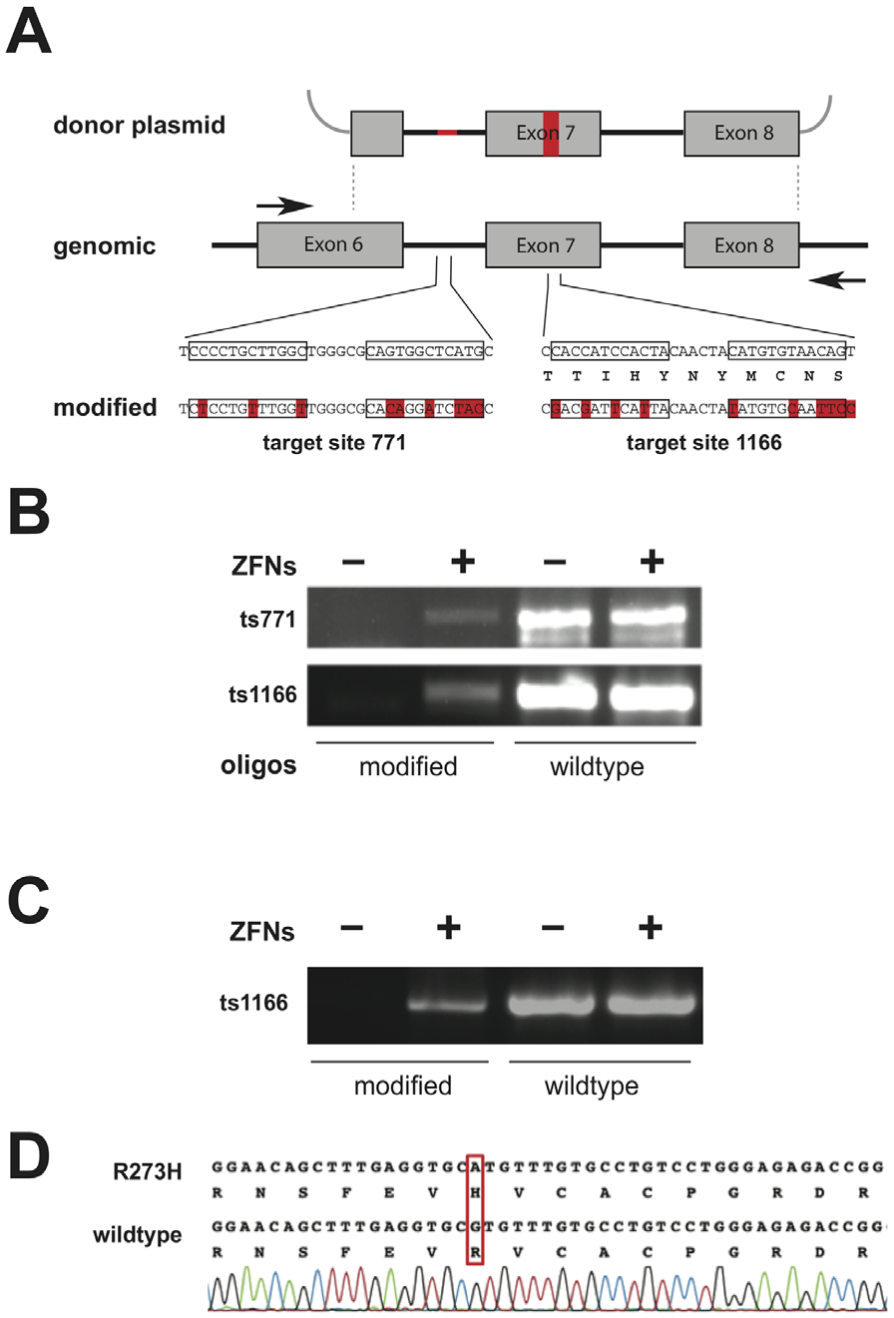
Targeted genomic recombination with p53 donor plasmid. (*A*) Schematic of the genomic p53 locus and the donor plasmid, with modified DNA sequences at z771 and z1166 target sites (“barcodes”; highlighted in red). Barcodes allow selective PCR but do not change the p53 protein sequence. Black arrows indicate primers for genomic PCR that hybridize to the chromosomal *p53* locus outside of the region corresponding to donor sequence. (*B*) Targeted donor plasmid recombination into the *p53* z771 and z1166 loci in HEK293T cells. ZFN-induced recombination is shown by semi-nested PCR, on purified external genomic PCR template, with forward primers that discriminate between wild-type and barcode sequence. (*C*) Targeted donor recombination at the *p53* z1166 locus in human SF268 glioblastoma cells. (*D*) Restoration of p53 wild-type sequence at codon 273 in ZFP-treated SF268 glioblastoma cells. The relative positions of the z1166 cutting site and codon 273 are indicated in Fig. 1B.

The donor plasmids contained modified sites in the target sites of the z771- and z1166-ZFNs, to avoid cutting of the donor plasmid by the respective ZFNs, and to allow detection of genomic recombination of the plasmid by PCR analysis (**Fig. 5**). The latter was achieved using external PCR primers to amplify the p53 genomic regions, followed by semi-nested PCR, with a forward primer specific for the modified DNA sequence (“barcode”), and an external genomic reverse primer. The modified barcodes in the exonic target z1166 were carefully-chosen silent mutations that do not alter the p53 amino acid sequence.

For analysis of genome editing, genomic DNA was prepared from a pool of treated HEK293T cells, 3–6 days after transfection. Targeted donor recombination at the *p53* locus was demonstrated by semi-nested PCR (**Fig. 5B**). Both ZFN-pairs were able to induce recombination of the donor plasmid with the chromosomal p53 gene, whereas control cells, transfected only with donor plasmid (and an empty PGK expression vector), did not show any sign of donor plasmid recombination. Although ZFN-specific recombination was seen with both repair matrices, the shorter exon 6–8 donor plasmid gave the clearest results because we were able to employ a particularly specific external genomic primer, just at the start of exon 6. Therefore this donor plasmid was mainly used in subsequent assays.

Next, we applied our z1166-ZFNs to induce the restoration of wt-p53 status in the human glioblastoma cancer cell line SF268, which harbors a single missense mutation (cgt→cat) at codon 273 in the core domain of p53 [59]. The SF268 cells were also transfected with PGK-driven z1166-ZFN expression vectors and a donor plasmid with wild type p53 sequence at codon 273. The treated cells were analyzed by PCR, as described for the HEK293T cells, and also showed site-specific recombination of the donor plasmid with the p53 gene, only when co-transfected with functional ZFNs (**Fig. 5C**).

As the point mutation in SF268 cells is located in exon 8, approximately 450 bp downstream of z1166 target site, PCR amplicons obtained with recombination-specific primers were subcloned by Topo-TA cloning; 10 clones were sequenced to check for downstream modification at the mutated R273H codon. All the clones showed a restoration of the p53 wild-type sequence at codon position 273. This indicated that homology-directed repair occurred >450 bp downstream of the ZFN-induced double-stranded break (**Fig. 5C**, bottom). This result is perhaps surprising, because 80% of gene conversion tracts in mammalian cells are expected to be within 100 bp of the double-stranded break [60]. Insertions can occur 400 bp away and further, albeit with much lower frequency [61], [62]. It was therefore possible that the PCR amplicons did not reflect independent events, and so we set out to measure the frequency of ZFN-induced homologous recombination.

### Measuring gense repair and non-homologous end-joining by deep sequencing

Next generation sequencing is an ideal tool to quantify the effects of ZFN on cells. Reads from genomic PCR-products can routinely give >20 million sequences (∼100 bp length) in a single run, and primer barcoding can be used to mix different samples together, allowing subsequent data deconvolution. We therefore developed a Solexa-Illumina method to sequence p53 locus genomic PCRs, at the site targeted by z1166. We thus measured the short insertions and deletions caused by non-homologous end-joining (NHEJ), after a nuclease-induced double-stranded break [25]. We also measured the rate of ‘wt’ sequence insertion from a ‘barcoded’ donor plasmid (with wild-type protein-coding sequence).

**Figure 6:**
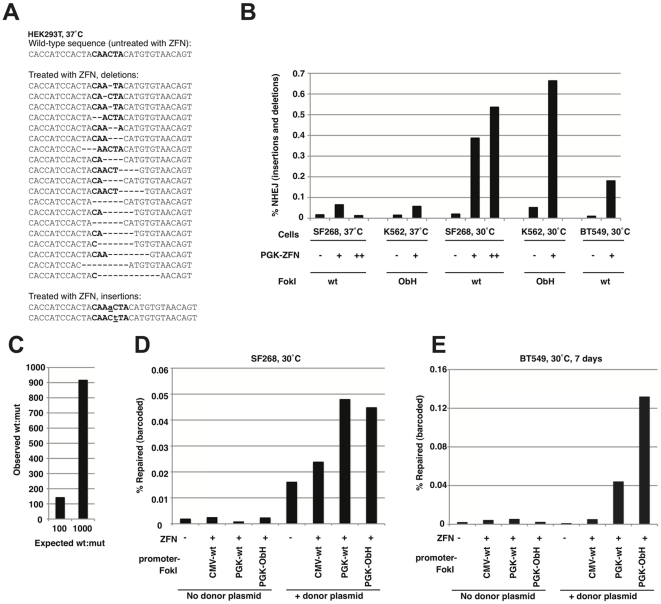
Solexa-Illumina deep sequencing of ZFN-treated cells. (*A*) Short 31bp reads detect ZFN-induced non-homologous end-joining (NHEJ) events in HEK293T cells. The *FokI* cutting region ( **CAACTA**) is indicated in bold, deletions are shown with “−” and insertions are underlined. (*B*) 104bp-read protocol: quantifying the rate of insertions and deletions induced by ZFN under a PGK promoter. SF268, K562 and BT549 cells were kept at 37°C or subjected to transient cold shock to increase NHEJ (30°C; [63]). ZFN plasmid amounts (0, 1 and 1.5 µg) are indicated by −, + asnd ++, respectively. Obligate heterodimer FokI mutants [57] are indicated where used (ObH). (*C*) Controls using wt∶mutant plasmids at ratios of 100∶1 and 1000∶1. After mixing, samples were treated identically as for other Solexa samples (3bp-barcode PCR, adapter ligation etc.). The proportion of wt∶mutant sequence in the Solexa output was then calculated. (*D*) Quantifying the rate of insertion of a barcoded (wt coding sequence) donor plasmid into the genomic p53 locus, in SF268 cells. CMV and PGK promoters were tested in combination with wt or ObH FokI nuclease domains. (*E*) A similar gene repair experiment in BT549 cells. Genomic DNA was collected 7 days after ZFN and donor plasmid transfection, to reduce background.

First, using 31bp reads, we observed NHEJ from ZFN in HEK293T cells (**Fig. 6A**). The method involved 2 rounds of PCR: one external genomic PCR and one internal PCR to introduce 3bp sequencing barcodes and an *MmeI* cleavage site (see **Methods S1**). *MmeI* digestion allowed sequencing-adapter ligation as close to the region of interest as possible but, as a result, the method was qualitative rather than quantitative. Nonetheless, the method showed that ZFN treatment was required to observe insertions and deletions around the genomic cutting site; these mutations can be useful for knocking out genes [38], [39].

Next, we carried out a series of experiments using an improved protocol: 104bp read length was achieved using a Solexa Genome Analyser IIx. After external genomic PCR, a second internal PCR introduced a 3bp sequencing barcode; the longer read length removed the need for *MmeI* digestion. Interestingly, we found that the slightly higher error rate of the newer HiSeq Solexa machine was suboptimal for this task, so the GA IIx was preferred. To achieve high-quality long reads with highly-similar PCR products, we found it was necessary to ‘spike’ the samples with random DNA (*phiX* DNA fragments; 50% of total input DNA). In each flow cell lane, after computationally filtering out *phiX* sequences, we were able to get around 5 million sequences in the correct orientation (∼50%). We were thus able to mix up to 8 sequencing barcodes per lane (each representing a different sample, under different conditions), resulting in around 600 000 reads each.

Processing the data for measuring NHEJ required two steps. First, the different barcoded samples were extracted using filters for any sequences containing a 9bp prefix (with the 3bp unique sequencing barcode) and a 9bp suffix (after the ZFP binding site)(see **Methods S1**). Second, to reduce random sequencing errors (proportional to read length), we filtered for the short ∼30bp region spanning the cutting site (sequences containing a new 6bp prefix-suffix; **Methods S1**). The percentage of sequences containing insertions or deletions was then calculated (**Fig. 6B**). By testing different cancer cell lines (SF268, K562 and BT549) we found that ZFN-dependent indels could be detected in all three, but were much more frequent at 30°C (transient cold shock) than at constant 37°C, as was recently reported [63]. Furthermore, we could observe NHEJ with both wt and obligate heterodimer FokI [57]. The increase in NHEJ signal with ZFN was up to 30-fold over background, indicating that next generation sequencing can be used reliably to measure this activity.

ZFN-driven gene repair was quantified in two human cancer cell lines (SF268, and BT-549). The cell lines were either transfected with z1166-ZFNs alone or with both ZFN and donor plasmid (to quantify ZFN-induced homologous recombination). First, the ability of the Solexa system to detect proportions of wt or mutant DNA was tested; plasmid samples were mixed at ratios of 1∶100 or 1∶1000, and were then processed as if they were genomic PCRs (**Fig. 6C**). The observed detection rate was indeed similar to that expected, despite the PCR amplification and adapter ligation steps during sample preparation. Next, a variety of constructs with different promoter and FokI nuclease variants were tested for their ability to induce homologous recombination (insertion of the donor plasmid sequence)(**Fig. 6D,E**). The best results were obtained with obligate heterodimer FokI nuclease [57], under a PGK promoter. Collecting the genomic DNA 7 days after the ZFN and donor plasmid transfection also helped to reduce background (Sigma Aldrich; Compo-Zr instructions). Although the absolute rates of homologous recombination are apparently quite low (∼0.1%), this is still an ∼100-fold improvement over background, indicating that the ZFN are functional at this locus.

In summary, we were able to use Y1H to engineer ZFN against p53 chromosomal targets and were able to show their activity to modify genomes at the selected loci.

## Discussion

This study describes the development of a new yeast-based selection tool for the rapid construction and optimization of paired 4-finger ZFN [19]. We developed the Y1H tool because we found that our usual phage display system [46] did not work with more than three fingers. Selections from 4-finger libraries resulted in spontaneously-truncated variants with fewer fingers, via in-frame homologous recombination in the bacterial host (data not shown). The truncated proteins were likely preferentially encapsidated, displayed and infected, and so 4-fingers are beyond the size limit that can be conveniently selected on capsid gene III. Despite trying different bacterial host strains, selection conditions, and even a PCR-gel-purification step in between selection rounds (to recover full-length clones), we were unable to overcome these issues. As classical phage display could not handle more than 3-fingers, we developed the yeast one-hybrid system, whose main advantage is that it uses PCR libraries directly and does not require a bacterial cloning step.

Our main motivation was to be able to screen relatively small libraries (<100 000 variants), with mutations spread over four or more fingers (the system works equally well for 6-finger proteins; **Fig. S3**). Small, targeted libraries can easily be rationally designed, given the 15 years of data on the zinc finger DNA-recognition code [15], [53]. Moreover, the Y1H system can also be used for affinity maturation of ZFP; libraries can be made by error-prone PCR and PCR-shuffling for this purpose, starting from a single ZFP design.

The use of yeast-based selection has certain advantages over phage display or prokaryotic expression systems (although the latter allow larger library sizes). For instance, the strategy allows the direct selection of peptides that are able to recognize DNA in vivo, without disrupting an eukaryotic cell. Thus, an element of screening for eukaryotic specificity and neutrality is added: the yeast genomic DNA, which is assembled into chromatin, is more repesentative of the final environment where the ZFN will be used. Nonetheless, the selection is for DNA binding and not ZFN cleavage; future engineering strategies could aim to select for cleavage activity and specificity directly. For example, the ZFN could cleave a target-DNA that is contiguous with a conditionally-lethal gene in yeast, such as Herpes simplex virus-thymidine kinase (HSV-TK) [64].

It should be noted that the quality of the screening is strongly dependent on the library design. In three cases (z771L, z1166L and R), the Y1H libraries produced functional ZFN that were much better than the original rational designs, around which the libraries were based. Indeed, in these cases, the single rational designs had no activity in the in vitro cleavage assay, whereas Y1H clones had demonstrable activity (**Fig. 3**). The exception was z771R, which was rationally designed and functioned so well that no improvements were obtained from Y1H. Typically, however, Y1H made the difference between a functional or non-functional ZFN.

We generated novel functional 4-finger nucleases against two sites located within the human p53 gene, in close vicinity to the mutation hotspots of p53 in cancers. The uniqueness of the targets in the genome was verified by a genome scanning algorithm that we developed (Table S1). This showed that the z771 and z1166 target sites are unique, and that the z1166 binding site had very few related targets in the human genome (only one 2bp mismatch and eight 3bp mismatches, for the full heterodimer site). z771 had slightly more related targets, but these are mostly in duplicated intronic sequences. Intron sites are likely to be more tolerant of indels from NHEJ, because no coding sequence is disrupted. Furthermore, intron sites can still be used for exonic gene repair, because homologous recombination can extend for hundreds of bases beyond the double-stranded break.

Initially, expression of our ZFN constructs was driven by a strong CMV promoter, but this was found to be suboptimal. It is possible that high expression levels of the nucleases were not well tolerated by the cells, probably leading to their elimination over time through accumulation of non-specific double strand breaks (**Fig. S2**) [56]. Alternatively, the CMV promoter expressing ZFN could compete with the CMV-driven GFP reporter, decreasing expression of both. For example, we observed that CMV-HcRed plasmid, used as a transfection marker, had reduced activity when co-transfected with other CMV-driven plasmids, but not with PGK-driven plasmids. After optimisation, i.e. finding the right promoter for ZFN expression (PGK), and adding a nuclear localization signal to the ZFN, we were able to get a good induction of GFP repair by both our anti-p53 ZFNs.

Using the Solexa protocol to quantify ZFN effects on the z1166 chromosomal locus, we confirmed that transient 30°C cold shock [63] improves the rates of NHEJ. The different cell lines each had different rates of NHEJ, and K562 had the highest. For gene repair, the best results were obtained using obligate heterodimer ZFNs [57], under a PGK promoter, and waiting for 7 days after transfection to reduce background from left-over donor plasmid. Although the apparent rates of homologous recombination are quite low (∼0.1%), the percentage of modified cells may actually be higher, since both alleles will not be modified in all cases. Moreover, for targeted mutation or repair experiments, the use of selection genes (e.g. puromycin resistance), combined with these low but workable recombination frequencies, should help to establish model cell lines or organisms.

We have demonstrated that our p53-specific ZFNs are functionally active chromosomally and can be used to mutate or restore wt-p53 status. Overall, this study has provided a Y1H tool to optimise ZFN, as well as functional anti-p53 ZFN for biotechnological applications.

## Materials and Methods

### Cell lines and culturing

The wt p53 cell line HEK293T was maintained in DMEM and the mutant p53 cell line SF268 (kindly provided by A. Carnero) was cultured in RPMI1640 at 37°C, in 5% CO_2_. All media were supplemented with 10% FCS, 100 units/ml penicillin and 100 µg/ml Streptomycin.

### Yeast one-hybrid selection of p53 zinc-fingers

Four-finger library cassettes were constructed from oligonucleotides, using a PCR based construction approach (library designs are listed in **Methods S1**). Two-finger units (F1-F2 and F3-F4) were built from two oligonucleotides by overlapping primer extension [54]. After amplification by PCR, introducing a BamHI site at the 3′-end of F1-F2 and a BglII site at the 5′-end of F3-F4, two-finger units were mixed, cut with BamHI and BglII and conditionally ligated. The resulting four-finger library cassettes (F1-F2-F3-F4) were amplified by PCR by using oligonucleotides which added 5′-and 3′-homology arms for the prey plasmid (sequences in **Methods S1**).

Target sequences were inserted into pHis2.1 bait plasmid (Clontech), using 22bp duplex DNA oligomers. Each pair of oligonucleotides was annealed to form duplex DNA, with EcoRI/SpeI compatible overhangs, and ligated into EcoRI/SpeI-cut vector pHis2.1.

pGADT7-Rec2 prey plasmid (Clontech Matchmaker One-Hybrid Library Construction and Screening Kit; Ref. 630304) was modified by removing a second HindIII site at 2351bp by site directed mutagenesis. The PCR libraries were then introduced into HindIII-linearized modified prey plasmid, by in-frame recombination in yeast strain Y187. The yeast were simultaneously heat-transformed with linearized prey plasmid, PCR library and bait plasmid, according to the manufacturer's instructions.

ZFPs with specific DNA-binding activities were recovered by plating the yeast transformations onto selective SD medium. Colonies growing on SD medium lacking histidine, tryptophan and leucine, but supplemented with 100mM or 150 mM 3-amino-1,2,4-triazole (3-AT), were replated on the same medium. ZFP genes were extracted from yeast by colony PCR (KOD polymerase; 2mM Mg; initial lysis at 98°C, 8 min) followed by a nested PCR to introduce a T7 promoter for protein expression in a TnT T7 Quick for PCR DNA kit (Promega). The coding sequence for the FokI-nuclease domain was fused in frame, via PCR, to create functional ZFNs (**Fig. S1**). All primers and conditions are indicated in **Methods S1**. The activity of the resulting ZFNs was verified by an *in vitro* DNA cleavage assay (see below).

### Zinc finger nuclease constructs and plasmids

ZFN coding sequences were cloned into BamHI/SalI cut plasmid pPGK.GZF1-N (gift from Toni Cathomen) [58]. To create ZFN of the high-fidelity obligate heterodimer type [57], we introduced point mutations in FokI using the QuickChange mutagenesis kit (Invitrogen). The nuclease expression vector pRK5.LHA-SceI, the repair plasmid pUC.Zgfp/Rex and the target plasmid pCMV.lacZsdGFP “0-0” have been described before [56]. The target plasmids pTARGET-771, pTARGET-1166 and palindromic target plasmids pTARGET-pts771L, pTARGET-pts771R, pTARGET-pts1166L, pTARGET-pts1166R were created by adding additional binding sites for the respective ZFNs. Two complementary synthetic 39-mer oligonucleotides were annealed and cloned into the *PacI* site of pCMV.lacZsdGFP “0-0” to generate each of the target sequences indicated in **Fig. 1B,C** and **Fig. 2A**. The ZFN target sites in each construct were verified by DNA sequencing.

### In vitro DNA cleavage assay

A 154bp linear target DNA was amplified by PCR from pTARGET plasmids with primers Target_seqL1 (5′-accagttggtctggtgtcaa-3′) and Target_seqR1 (5′-ctgaacttgtggccgtttac-3′). DNA templates for in vitro expression of ZFNs were amplified by PCR from positive yeast colonies or from ZFN expression vectors using primers T7kozak_FWD (5′-tcgagtaatacgactcactatagggagaaacaccatagattgccatggccgagcgccccttc-3′) and UnivQQR_R_NotI (5′-aaggaaaaaagcggccgcaaaaggaaaaggatcctcattaaaagtttatc-3′). All PCR reactions were purified with the QiaQuick PCR Purification Kit (Qiagen).

The ZFNs were expressed from 100ng T7-PCR templates, using the TnT-Quick coupled transcription-translation system (Promega), according to the manufacturer's protocol, except that ZnCl_2_ was added to a final concentration of 4 mM. To analyze ZFN activities, TnT reactions were mixed with 200 ng of target DNA and diluted with *FokI* cleavage buffer to a final concentration of: 20 mM Bis-Tris-propane pH 7.0, 100 mM NaCl, 5mM MgCl_2_, 0.1 mM ZnCl_2_, 5 mM DTT, 1.8% (vol/vol) glycerol, 20 ug/ml poly-d(I-C), 0.1 mg/ml BSA. After incubation for 5–6 hrs at 30°C, the reactions were cleaned with a PCR Purification Kit (Qiagen) or, alternatively, were treated with 1 µl RNase A (10 mg/ml, Qiagen) for 30 minutes at 37°C and 1 µl Proteinase K (20 mg/ml, Qiagen) for 1 hour at 37°C. Samples were then analysed on a 1.8% agarose gel, with ethidium bromide staining.

### Episomal gene repair assay

For episomal gene repair, we typically followed the protocol described in [56]. Briefly, HEK293T cells in 24-well plates were transfected by Lipofectamine 2000 (Invitrogen) with 100 ng of target plasmid, 300ng of repair plasmid pUC.Zgfp/REx or repair control pUC.REx, and 100ng of PGK-driven ZFN expression vectors, I-SceI (pRK5.LHA-SceI) or control vector pUC19. The amount of DNA was kept constant by adding pUC19 to 500ng. After 48 hrs, 5×10^5^ cells were analyzed by flow cytometry (FACSCanto, BD Bioscience) to determine the percentage of EGFP- and REx-positive cells. Statistical significance was determined using the Student t-test. Error bars in figures are one standard error based on three biological replicates.

### PCR analysis of genomic recombination of p53-donor plasmid

2×10^6^ K562 cells were transfected with 5 µg of each ZFN expression construct, and 10 µg of the donor plasmid, using the single-cuvette format of the Nucleofector Kit V (Lonza), according to the manufacturer's protocol. HEK293T, SF268, and BT-549 cell lines were transfected in 6-well plates by Lipofectamine 2000 (Invitrogen), with 5 µg repair matrix donor plasmid, and 2 µg of ZFN expression vectors, or empty control vector. An EGFP expression vector (0.5 µg) was cotransfected in all the samples (except K5652 cells) to identify transfected cells. Six days after transfection, 1×10^5^ GFP-positive cells per sample were collected by fluorescence activated cell sorting (FACS) and genomic DNA was isolated from cells with the DNA Blood and Tissue Kit (Qiagen). To detect homologous recombination in the p53 gene, we subjected 30–300 ng genomic DNA to PCR with high fidelity enzymes KOD Hot Start (Novagen) or Accuprime Taq DNA Polymerase (Invitrogen). PCR products were column purified with the PCR purification kit (Qiagen) and eluted in 30 µl H_2_0. A fraction of the resulting amplicons was amplified by nested PCR with Taq polymerase using primers designed to discriminate between wild-type and integrated modified p53 sequence. Nested PCR amplicons were resolved on a 1.5% agarose gel and visualized by ethidium bromide staining. All primers and conditions are indicated in **Methods S1**.

### Quantification of non-homologous end-joining and homologous recombination events

The frequency of targeted gene modification in ZFN-treated pools of cells was determined by Illumina's Solexa deep sequencing platform. Transfections, genomic DNA preparation and PCR reactions were performed as described above. PCR products were excised from agarose gels, to avoid contamination with transfected donor plasmid, were column purified with a PCR purification kit (Qiagen) and eluted in 30 µl H_2_0. 10 ng of the PCR products were amplified by nested PCR with KOD HiFi polymerase, with Solexa primers flanking the 1166 target region (∼90bp up- or down-stream). The primers contained 3bp barcodes in order to distinguish between individual samples in a pooled Solexa lane. PCR amplicons were column purified for sequencing adapter ligation with Illumina protocols.

### Illumina sequencing and basecalling

PCR products that contained single-read Solexa adapters on either end were mixed 1∶1 with a phiX Solexa library (Illumina). Single-read v4 flowcells for the Genome Analyzer were used. After loading DNA at a concentration of 7 pM per flow cell lane, clusters were generated in the Illumina cluster station according to the recommendation of the manufacturer. Sequencing was performed on the Illumina Genome Analyzer IIx with TrueSeq SBS v5 sequencing chemistry, using a 104 cycle recipe. Basecalling was performed using SCS2.8. Primers and conditions and computational filters used for data processing are indicated in **Methods S1**.

## Supporting Information

Figure S1 **Experimental scheme to validate yeast one-hybrid clones.** Assembly PCR was used to recover zinc finger sequences from positive yeast colonies and to fuse them to a FokI nuclease domain and T7 promoter, for in vitro transcription-translation expression (TnT). Clones marked with an asterisk showed clear cleavage activity of the target DNA (palindromic target site). These positives were subcloned for further verification.
(TIF)

Figure S2 **ZFN-associated toxicity assay.** Flow cytometry data for HEK293T cells transfected with the indicated constructs and stained with antibodies against gH2A.X (top) or unspecific staining-control antibodies (bottom). The columns show the percentage of gH2A.X-positive cells normalized for transfection efficiency. Overall, pPGK constructs had lower toxicity than pCMV constructs, although the meganuclease I-SceI had even lower toxicity. Etoposide is a toxic positive control.
(TIFF)

Figure S3 **Six finger peptides engineered by Y1H for the GFP DNA sequence.** (A) Canonical model of ZFP binding, where primary DNA contacts (arrows) are from four positions on each zinc finger alpha helix (circled, −1, 2, 3 and 6). Contacts shown are for the EGFP gene-binding construct, ZFP_GFPb249. (B) List of ZFPs engineered against the EGFP coding sequence (ZFP_GFPb249). The alpha helices are shown aligned to the DNA bases they would contact according to the canonical model. Note that zinc finger proteins (N-C) bind antiparallel to their primary contacting DNA strand (3′-5′). (C) Gel shift assays on ZFPs expressed in vitro from T7-promoter PCR products show that the 2 ZFPs bind their DNA targets.
(TIFF)

Table S1 **Putative off-target sites.** The number of occurrences (in the human genome) of sequences related to the target sequence were counted with a computer script written in C. For example, there are 35 sequences with 2 bases different from the full z771 zinc finger binding site (bs771). Overall, the z1166 binding site has fewer related targets in the human genome. bs = binding site. pts = palindromic target site. The different left- and right-finger binding sites are highlighted in bold or normal font, respectively.
(DOCX)

Methods S1 **Supplementary methods and sequences.** This document contains sequence templates and instructions for inserting zinc finger DNA sequences into the yeast 1-hybrid template. Strategies for selective codon randomisation are discussed. The sequences for the zinc finger libraries, final clones and donor plasmid sequences used in this study are also provided. Protocols for Solexa sequencing of ZFN genomic targets are described, as well as protocols for measuring double-stranded DNA breaks.
(DOC)
