# Supplementary figures and images for: p53 Gene Repair with Zinc Finger Nucleases Optimised by Yeast 1-Hybrid and Validated by Solexa Sequencing
Source: PLoS One. 2011 Jun 9;6(6):e20913. doi: 10.1371/journal.pone.0020913 (PMC3111460; doi:10.1371/journal.pone.0020913)

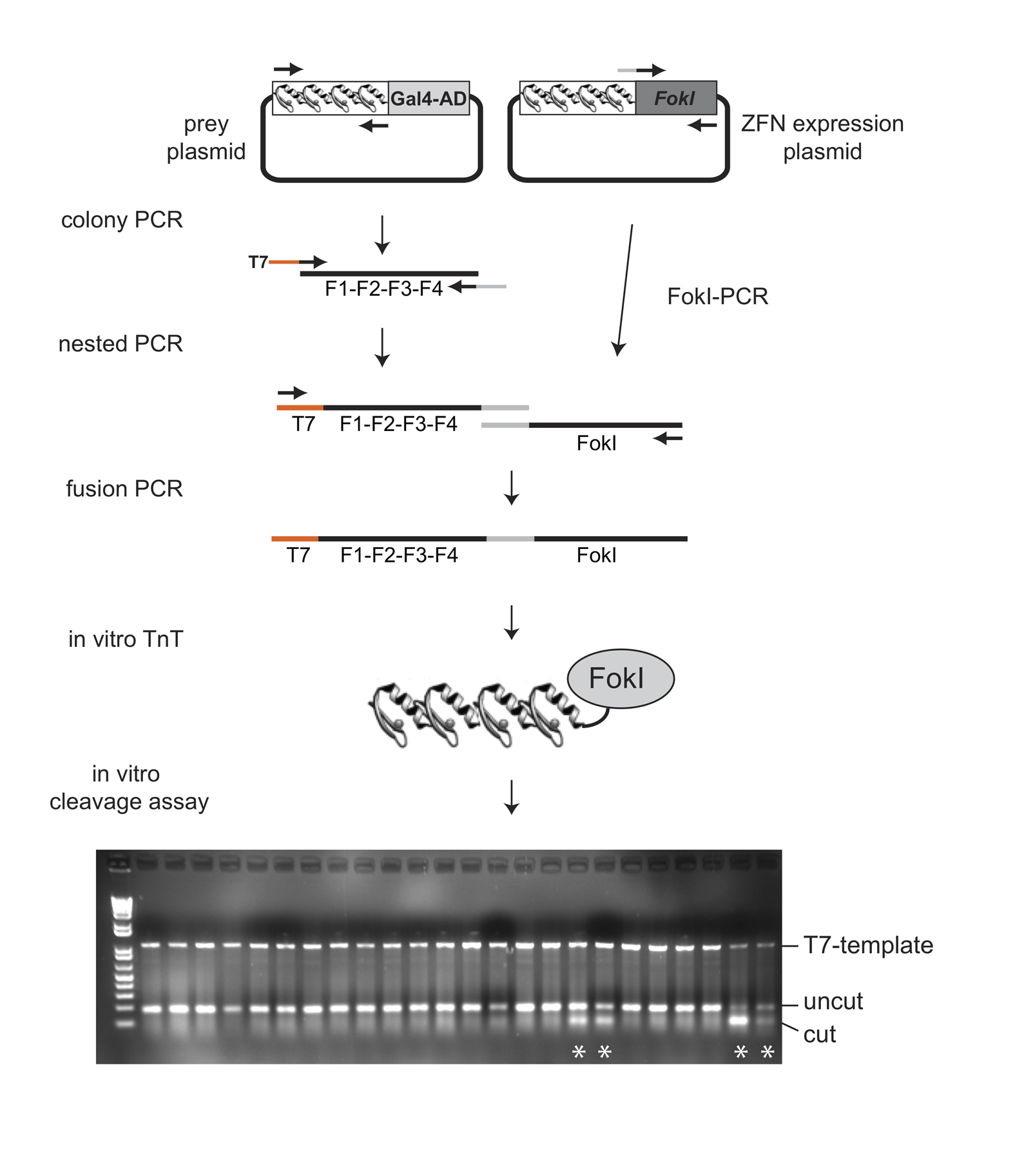

Supplement: Figure S1 — Experimental scheme to validate yeast one-hybrid clones. Assembly PCR was used to recover zinc finger sequences from positive yeast colonies and to fuse them to a FokI nuclease domain and T7 promoter, for in vitro transcription-translation expression (TnT). Clones marked with an asterisk showed clear cleavage activity of the target DNA (palindromic target site). These positives were subcloned for further verification. (TIF) [file pone.0020913.s001.tif]

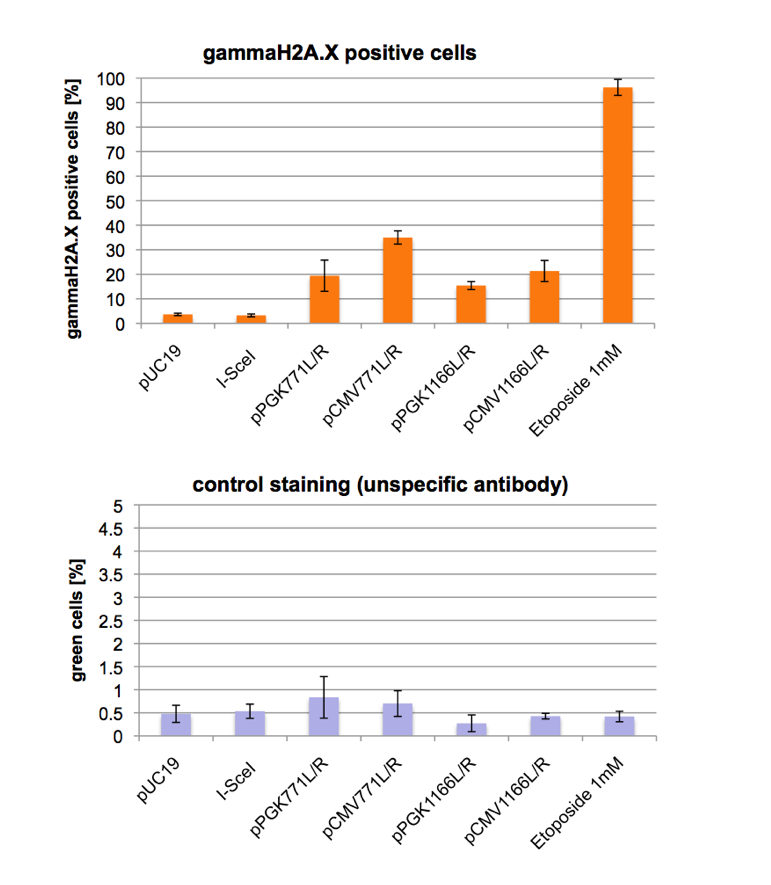

Supplement: Figure S2 — ZFN-associated toxicity assay. Flow cytometry data for HEK293T cells transfected with the indicated constructs and stained with antibodies against gH2A.X (top) or unspecific staining-control antibodies (bottom). The columns show the percentage of gH2A.X-positive cells normalized for transfection efficiency. Overall, pPGK constructs had lower toxicity than pCMV constructs, although the meganuclease I-SceI had even lower toxicity. Etoposide is a toxic positive control. (TIFF) [file pone.0020913.s002.tif]

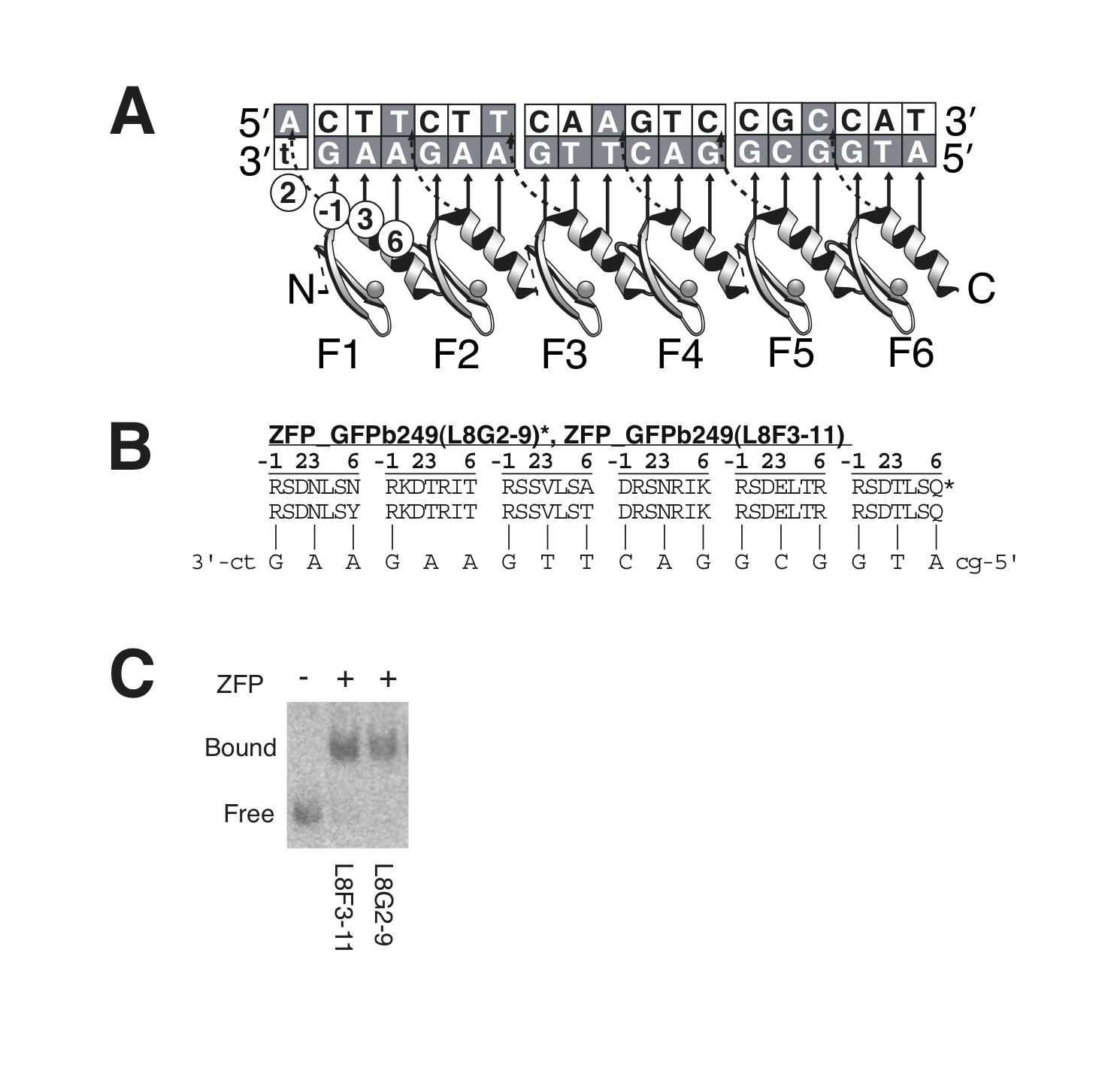

Supplement: Figure S3 — Six finger peptides engineered by Y1H for the GFP DNA sequence. (A) Canonical model of ZFP binding, where primary DNA contacts (arrows) are from four positions on each zinc finger alpha helix (circled, −1, 2, 3 and 6). Contacts shown are for the EGFP gene-binding construct, ZFP_GFPb249. (B) List of ZFPs engineered against the EGFP coding sequence (ZFP_GFPb249). The alpha helices are shown aligned to the DNA bases they would contact according to the canonical model. Note that zinc finger proteins (N-C) bind antiparallel to their primary contacting DNA strand (3′-5′). (C) Gel shift assays on ZFPs expressed in vitro from T7-promoter PCR products show that the 2 ZFPs bind their DNA targets. (TIFF) [file pone.0020913.s003.tif]
