# Supplementary material for: p53 Gene Repair with Zinc Finger Nucleases Optimised by Yeast 1-Hybrid and Validated by Solexa Sequencing
Source: PLoS One. 2011 Jun 9;6(6):e20913. doi: 10.1371/journal.pone.0020913 (PMC3111460; doi:10.1371/journal.pone.0020913)
Supplement: Table S1 — Putative off-target sites. The number of occurrences (in the human genome) of sequences related to the target sequence were counted with a computer script written in C. For example, there are 35 sequences with 2 bases different from the full z771 zinc finger binding site (bs771). Overall, the z1166 binding site has fewer related targets in the human genome. bs = binding site. pts = palindromic target site. The different left- and right-finger binding sites are highlighted in bold or normal font, respectively. (DOCX) [file pone.0020913.s004.docx]

| **Target sequence** |  | **0bp** | **1bp** | **2bp** | **3bp** | **4bp** |
| --- | --- | --- | --- | --- | --- | --- |
| pts771L | cctCATGAGCCACTGtgggcgCAGTGGCTCATGcct | 0 | 0 | 0 | 2 | 25 |
| pts771R | cct**CCCCTGCTTGGC**tgggcg**GCCAAGCAGGGG**cct | 0 | 0 | 0 | 0 | 14 |
| bs771 | cct**CCCCTGCTTGGC**tgggcgCAGTGGCTCATGcct | 0 | 7 | 35 | 457 | 1956 |
| pts1166L | tacCTGTTACACATGcaactaCATGTGTAACAGttc | 0 | 0 | 0 | 1 | 14 |
| pts1166R | tac**CACCATCCACTA**caacta**TAGTGGATGGTG**ttc | 0 | 0 | 0 | 0 | 3 |
| bs1166 | tac**CACCATCCACTA**caactaCATGTGTAACAGttc | 0 | 0 | 1 | 8 | 20 |
