# Supplementary material for: p53 Gene Repair with Zinc Finger Nucleases Optimised by Yeast 1-Hybrid and Validated by Solexa Sequencing
Source: PLoS One. 2011 Jun 9;6(6):e20913. doi: 10.1371/journal.pone.0020913 (PMC3111460; doi:10.1371/journal.pone.0020913)
Supplement: Methods S1 — Supplementary methods and sequences. This document contains sequence templates and instructions for inserting zinc finger DNA sequences into the yeast 1-hybrid template. Strategies for selective codon randomisation are discussed. The sequences for the zinc finger libraries, final clones and donor plasmid sequences used in this study are also provided. Protocols for Solexa sequencing of ZFN genomic targets are described, as well as protocols for measuring double-stranded DNA breaks. (DOC) [file pone.0020913.s005.doc]

**Methods S1 - Supplementary methods and sequences**

**p53 gene repair with zinc finger nucleases optimised by yeast 1-hybrid and validated by Solexa sequencing**

Frank Herrmann1, Mireia Garriga-Canut1, Rebecca Baumstark1, Emmanuel Fajardo-Sanchez1, James Cotterell1, André Minoche2,3, Heinz Himmelbauer3 and Mark Isalan1

^1^EMBL/CRG Systems Biology Research Unit, Centre for Genomic Regulation (CRG) and UPF, 08003 Barcelona, Spain. ^2^Max Planck Institute for Molecular Genetics, Ihnestr. 63-73, 14195 Berlin, Germany. ^3^Ultrasequencing Unit, Centre for Genomic Regulation and UPF, 08003 Barcelona, Spain.

**Contents**

**Page**

**1. Template for Y1H............................................................................. 2**

**2. Rationale and protocol for designing a Y1H library..................... 3**

**2.1 Step 1. ZFP design....................................................... 3**

**2.2 Step 2. Fitting to Y1H template ................................. 3**

**2.3 Step 3. Randomisation ................................................ 4**

**2.3.1 Codons for ZFP Randomisation............ 5**

**2.3.2 Notes on choices for randomisation .... 6**

**2.3.3 Worked example: F2 of z1166L .......... 7**

**2.4 Step 4. DNA synthesis................................................. 7**

**2.5 Step 5. Yeast transformation ..................................... 7**

**3. Four-finger libraries for p53 targets ............................................ 8**

**4. Donor plasmid sequences .............................................................. 12**

**5. Yeast and genomic PCRs .............................................................. 13**

**6. Solexa deep sequencing protocols ................................................ 15**

**6.1 Step 1. Genomic PCR .............................................. 15**

**6.2 Step 2. Sequencing barcode PCR ............................ 15**

**6.2.1 Protocol with MmeI digestion............. 16**

**6.2.2 Protocol without MmeI digestion........ 16**

**6.3 Step 3. Data processing.............................................. 17**

**7. Measurement of double-stranded breaks in ZFN-treated cells.. 18**

**8. DNA and protein sequences of zinc finger-FokI constructs........ 19-20**

**Supplementary Methods**

**1. Template for Y1H**

Below is an example of one ZFP PCR library, together with homology arms for direct yeast transformation according to the scheme in main text Fig. 2. Any in-frame zinc finger sequence can be inserted into the Matchmaker Y1H system using this template. The template below is based on 2 x 2-finger units of F2-F3 of the Zif268 sequence [66]. Each pair of 2-finger units is separated by a longer TGSERP linker [65]. The final linker, (QNKKQLVKSEL) is a mixture of the Sp1 ZFP C-terminal liner (QNKK) and the FokI leader sequence (QLVKSEL) and is adapted from [16]. DNA-recognition helices are selectively randomised. See also main Fig. 2C. The rationale behind library randomisation is explained on the following pages.

**>Library z1166L**

CAATATTTCAAGCTATACCAAGCATACAATCAACTCCAAGCTTACCATGGCCGAGGAACGCCCCTTCCAGTGTCGCATCTGTATGCGCAATTTCAGCCGCAGCGACAACCKGANMGWGCACATTCGCACGCACACCGGCGAAAAGCCCTTTGCCTGTGACATTTGTGGGCGCAAGTTCGCGCAGANMGCCAACCKGANMAMGCATACCAAAATTCACACCGGATCTGAGCGCCCGTTTCAGTGCAGGATTTGCATGAGGAACTTCTCCCGGTCCGACGCCCKGANMCGCCATATCAGGACCCATACCGGGGAGAAACCTTTCGCGTGCGATATCTGCGGGAGGAAATTCGCCAACANMAGCAACCKGANMGWGCACACGAAGATCCATCAGAACAAGAAGCAACTAGTCAAAAGTGAACTAAGCTTTGCAAAGATGGATAAAGCGGAATTAATTCCCGAGCCT

**Key:**

1. left homology arm (compatible with linearized prey plasmid pGADT7-Rec2)

2. HindIII sites

3. ACCATGG = Kozak consensus start codon

4. zinc finger library sequence

5. Underlined are the combinatorially randomized sequences for DNA-recognition α-helices.

6. peptide linker towards GAL4-AD

7. right homology arm (compatible with linearized prey plasmid pGADT7-Rec2)

**From Methods:**

The resulting four-finger library cassettes (F1-F2-F3-F4) were amplified by PCR by using oligonucleotides adding 5'-and 3'-homology arms and subsequently introduced into the HindIII-linearized modified prey plasmid pGADT7-Rec2 (second HindIII site at 2351bp was removed by site directed mutagenesis) of Clontech’s Matchmaker One-Hybrid Library Construction and Screening Kit (Ref 630304) by in-frame recombination in yeast strain Y187.

**Primers to remove unwanted extra HindIII Restriction Site in pGADT7-Rec2:**

>HIND3_Frw

TACGAGGGCTTATTCAGAAcCTTTGGACTTCTTCGCCAG

>HIND3_Rev

CTGGCGAAGAAGTCCAAAGgTTCTGAATAAGCCCTCGTA

PCR Settings:

(97˚C (30s), 46˚C (30s), 68˚C (5 min)) x 5

(97˚C (30s), 68˚C (5 min)) x 15

---

Use DpnI digestion prior to bacterial transformation to remove plasmid template.

**2. Rationale and protocol for designing a Y1H library**

**2.1 Step 1. ZFP design**

The first step is to obtain a ZFP design for optimisation. This can be done by rational design, based on the ZFP literature, or we would recommend using the ZiFiT webserver from the Zinc Finger Consortium:

http://zifit.partners.org/ZiFiT/ChoiceMenu.aspx

This conveniently matches ZFPs to DNA sites and provides ZFP-coding DNA sequences.

---

**2.2 Step 2. Fitting to Y1H template**

Having built and tested a ZFP and found its activity to be insufficient for purpose, the next step is to fit it into the framework on the previous page. We would suggest using this template:

**DNA:**

M A E E R P

CAATATTTCAAGCTATACCAAGCATACAATCAACTCCAAGCTTACCATGGCCGAGGAACGCCCC

-insert your ZFP coding sequence here, starting with F or Y (the first ZF hydrophobic residue) and ending in the final H-

CAGAACAAGAAGCAACTAGTCAAAAGTGAACTAAGCTTTGCAAAGATGGATAAAGCGGAATTAATTCCCGAGCCT

**Protein:**

MAEERP

FQCRICMRNFSKKDHLHRHTRTHTGEKP

FQCRICMRNFSQRSDLTRHLRTHTGEKP

FQCRICMRNFSQSGTLTRHLKTH

QNKK QLVKSEL SFAKMDKAELIPEP

Sp1- FokI- homology arm

linker linker

The example shown above is a 3-finger sequence from ZiFiT's CoDA design tool. End the zinc finger sequence with the last finger's final H-residue, next to the linkers for Sp1-FokI and the homology arm. Note that the linker between the final H and FokI can be varied to allow different spacers in the FokI cutting site, in the final zinc finger nuclease construct:

H Linker FokI Gap between ZFPs Source

H LRGS QLVKSEL 5-6bp (Joung)

H QNKK QLVKSEL 6bp (Carroll)

H TGQKD QLVKSEL 7bp (Porteus)

**2.3 Step 3. Randomisation**

Having designed your ZFP framework, the main decision is over which residues to randomise. Most ZFP libraries focus on randomising the residues in the DNA-binding α-helix, underlined above (e.g. KKDHLHR). There are 7 positions, numbered conventionally as -1, +1, +2, +3, +4, +5, +6 (relative to the start of the α-helix).

Randomisation is always a compromise between library size and binding potential. NNN codons, for example, give very large, comprehensive libraries but suffer from unwanted stop codons and very sparse appropriate DNA-binding residues. NNS codons are sometimes used to remove 2/3 of stop codons and reduce library sequence diversity, while still coding all amino acids. Error prone PCR is best suited for examples where just a few mutations, close to the original design, will give a large benefit.

In practice, we have found that small targeted libraries, with selectively-randomised codons, are a good compromise between library size and DNA-binding potential. We use the codons listed on the next page, and the choices are based on which amino acids work best at certain positions (mainly from previous phage display data). The codons highlighted in **bold** are preferred options, but the user must always choose between library size and library binding potential.

KEY:

**IUPAC Base code**

letter=DNA bases coded

R=AG

Y=CT

M=AC

K=GT

S=CG

W=AT

B=CGT

D=AGT

H=ACT

V=ACG

**2.3.1 Codons for ZFP Randomisation**

(Preferred options in **bold**)

Codon Helix-position DNA-sequence-degeneracy

| | (Potential |

| Amino acids | DNA-bases-bound) |

| | | | |

**ARG=KR**.......**-1,+6(G,T)**.................**2**

**AMC=NT**.......**-1,+3(A,T)**.................**2**

**RMC=NTAD**.....**-1,+3(A,T,C)**...............**4**

**CAS=QH**.......**-1(A,T,C)**..................**2**

MAC=NH.......-1(A,T,C)..................2

GHC=VDA......-1,+3(T,C,(N)).............3

RAC=DN.......-1(C,A,T)..................2

GWC=DV.......-1,+3(C,T).................2

GMC=AD.......-1,+3(T,C), +2(N)..........2

SAC=HD.......-1(T,C),+3(C,G)............2

GMS=ADE......-1,+3(T,C,(N)).............4

GVS=GADE ....-1,+3(C,(N))...............6

GHS=VADE.....-1,+3,+6(T,C,(N))..........6

RRK=NDESRKG..-1,+6(A,C,G,T,(N)).........8

**AGC=S**........**+1**.........................**1**

**ANM=RSKNTI**...**+1,+4,+5**...................**8**

**GMC=AD**.......**+2(N)**......................**2**

**RRC=DGNS**.....**+2(N), +3(A,T,C)**...........**4**

ARC=SN.......+2(N)......................2

KMC=YSAD.....+2(N)......................4

MRC=RHNS.....+2(N)......................4

VRC=RHSNGD...+2(N),-1,+3(N).............6

SDG=RQLGEV...+2(N)......................6

**RMC=TNAD**.....**+3,-1(A,T,C)**...............**4**

**CAC=H**........**+3(G)**......................**1**

GYC=AV.......+3(T,C)....................2

RCC=AT.......+3(T,C(N)).................2

ASC=ST.......+3(T,C)....................2

ASS=STR......-1,+3,+6 (G,T,C)...........4

ARS=SNKR.....-1,+3,+6 (N)...............4

AVS=SNKRT....-1,+3,+6 (N)...............6

ANS=SNKRTMI..-1,+3,+6 (N)...............8

Non-DNA-binding positions (+1,+4,+5):

**CKG=LR**.......**+4(N)**......................**2**

CDC=LRH......+4(N)......................3

**AWA=IK**.......**+5(N)**......................**2**

AHA=KIT......+5(N)......................3

AHM=KITN.....+5(N)......................6

**ANM=RSKNTI**...**+1,+4,+5**...................**8**

ABS=MIRST....+1,+4,+5...................6

**AMG=KT**.......**+6(G,T), +5(N)**.............**2**

**GWG=EV** ......**+6(C,T)**....................**2**

**TAC=Y**........**+6(A)**......................**1**

**CAG=Q**........**+6(A)**......................**1**

**CRG=QR**.......**+6(A,G)**....................**2**

**SAG=QE**.......**+6(A,C)**....................**2**

**DAC=YND** .....**+6(A,C)**....................**3**

MAG=QK.......+6(A,G)....................2

RYA=ATVI.....+6(T,C,(N))................4

GHG=EAV......+6,+3(T,C,(N)).............3

MAM=NQKH.....+6(A,G,T)..................4

RMG=TKAE ....+6(T,G,C,(N))..............4

RMK=TKAEDN ..+6(T,G,C,A(N)).............8

**2.3.2 Notes on choices for randomisation**

To vary position **-1**, see: Proc Natl Acad Sci U S A. 1994 Nov 8;91(23):11168-72. (or many other groups' publications, e.g. Barbas, Kim, Berg, Pabo, etc.). If in doubt, put:

**ARG=KR(bind G,T) or RMC=NTAD(bind A,T,C)**

To vary position **+1** (non DNA binding), use **ANM=RSKNTI or AGC=S**

To vary position **+2**, see Proc Natl Acad Sci U S A 94, 5617-21 (1997), Biochemistry 37, 12026-33 (1998). Position 2 often overlaps with the preceding finger.If in doubt, put:

**GMC=AD or RRC=DGNS**

To vary position **+3**, see: Proc Natl Acad Sci U S A. 1994 Nov 8;91(23):11168-72. (or many other groups' publications, e.g. Barbas, Kim, Berg, Pabo, etc.). If in doubt, put:

**RMC=TNAD(bind A,T,C) or CAC=H(bind G)**

To vary position **+4** (non DNA binding), use **CKG=LR**

To vary position **+5** (non DNA binding), use **ANM=RSKNTI or AMG=KT**

To vary position **+6**, see: Proc Natl Acad Sci U S A. 1994 Nov 8;91(23):11168-72. (or many other groups' publications, e.g. Barbas, Kim, Berg, Pabo, etc.). If in doubt, put:

**AMG=KT(bind G,T);GWG=EV(bind C,T);CRG=QR(bind A,G);TAC=Y(bind A);CAG=Q(bind A).**

The above choices are based on canonical binding modes and there are many examples where unexpected combinations of amino acids give high-affinity, specific DNA-binding. If in doubt, randomise more; even though this will lead to library undersampling, there may still be functional candidates found.

For illustration of this randomisation strategy, see Nature Biotechnology 19, 656-60 (2001).

**2.3.3 Worked example: F2 of z1166L**

Original design, shown aligned to DNA recognition site

(fingers bind N-C, 3'-5'):

3'-a a t-5'

| | |

N-QKANRTK-C

Randomisation:

QKANRTK

R LKT

S R

N S

T N

I I

Helix Codons**:**

CAG ANM GCC AAC CKG ANM AMG

Result after Y1H:

QNANRNT

Overall, z1166L only worked in a DNA-cleavage assay after Y1H optimisation, showing the value of the system.

**2.4 Step 4. DNA synthesis**

Having designed the final cassette with semi-randomised codons and appropriate homology arms for Y1H, order the library DNA cassette from a gene synthesis company (e.g. Genscript, DNA2.0 etc.)

**2.5 Step 5. Yeast transformation**

Adapted from Clontech’s Matchmaker One-Hybrid Library Construction and Screening Kit (Ref 630304). See their Manual for quantities and scales.

Mix the ZFP library PCR (with the appropriate homology arms) together with HindIII-linearized modified prey plasmid pGADT7-Rec2 (second HindIII site at 2351bp was removed by site directed mutagenesis), and the bait plasmid (pHis2.1, which must have one cloned ZFP-DNA-binding site in the EcoRI/SpeI sites). Transform this mixture into yeast using Clontech’s Matchmaker One-Hybrid Library Construction and Screening Kit (Ref 630304). In-frame recombination in yeast strain Y187 results in a complete ZFP prey library (see also Fig 2 and Methods). Plate and re-plate colonies on SD medium lacking histidine, tryptophan and leucine, but supplemented with 150 mM 3-amino-1,2,4-triazole (3-AT). Recover potential positives by PCR (Section 5., below).

**3. Four -finger libraries for p53 targets**

The 4-finger libraries designed here were made by oligo overlap primer fill-in (see Methods), followed by restriction digestion and ligation to make concatenated, 4-finger constructs. However, with recent advances in DNA synthesis, it is much more convenient to order such libraries from a DNA synthesis company, so only final library cassettes are listed.

Below, protein recognition helices (e.g. N-RSSSLTN-C) are shown aligned to target DNA recognition sequences (3'-5'). The randomised amino acids, and oligos encoding them, are indicated below. n=library size (synonymous codons are counted separately).

**z771L4**

3' g t a c t c g g t g a c 5'

| | || | | | | | | | |

RSSSLTN ARSSHIE RSGHLKT RSDNRTA

**R TRKQ SR LK RT LKT**

**K K KK R V**

**N H NN I**

**T TT**

**I II**

n: 8 2224 88 32 22 224 = ~6.3 million

(synonymous codons counted separately)

**>Library z771L4**

CAATATTTCAAGCTATACCAAGCATACAATCAACTCCAAGCTTACCATGGCCGAGGAACGCCCCTTCCAGTGTCGCATCTGTATGCGCAATTTCAGCCGCANMTCCASCCKGAMGMAMCACATTCGCACGCACACCGGCGAAAAGCCCTTTGCCTGTGACATTTGTGGGCGCAAGTTCGCGGCCANMANMTCCCDCAWAGAACATACCAAAATTCACACCGGATCTGAGCGCCCGTTTCAGTGCAGGATTTGCATGAGGAACTTCTCCCGCTCCGGCCACCKGAMGACCCATATCAGGACCCATACCGGGGAGAAACCTTTCGCGTGCGATATCTGCGGGAGGAAATTCGCCCGCAGCGACAACCKGAMGRYACACACGAAGATCCATCAGAACAAGAAGCAACTAGTCAAAAGTGAACTAAGCTTTGCAAAGATGGATAAAGCGGAATTAATTCCCGAGCCT

Result: **RNSSLTN ATNSLIE RSCHLKT RSDNLKT (C=random mutation)**

z771L only worked after Y1H optimisation (Fig 3.). Therefore the library worked even though it was undersampled.

**z771R**

3’ g g g g a c g a a c c g 5’

| | | | | | | | | | | |

RSSHLSR RNDNRKE RSSNLSQ DNSDRIR

**K K ASL V K NTR E N V KK**

**G A**

**D D**

n: 2 2 242 2 2 24 2 2 2 22 = 65,536

**>Library z771R**

CAATATTTCAAGCTATACCAAGCATACAATCAACTCCAAGCTTACCATGGCCGAGGAACGCCCCTTCCAGTGTCGCATCTGTATGCGCAATTTCAGCARGAGCAGCCACCTGAGCCGCCACATTCGCACGCACACCGGCGAAAAGCCCTTTGCCTGTGACATTTGTGGGCGCAAGTTCGCGARGAACGMCRRCCKGAAGGWGCATACCAAAATTCACACCGGATCTGAGCGCCCGTTTCAGTGCAGGATTTGCATGAGGAACTTCTCCARGAGCARCRMCCKGAGCSAGCATATCAGGACCCATACCGGGGAGAAACCTTTCGCGTGCGATATCTGCGGGAGGAAATTCGCCRACAACAGCGWCCGCAWAARGCACACGAAGATCCATCAGAACAAGAAGCAACTAGTCAAAAGTGAACTAAGCTTTGCAAAGATGGATAAAGCGGAATTAATTCCCGAGCCT

Result: tested in yeast but not better than rationally designed 771R_mod (which is already active in DNA cleavage assays):

**RSSHLSR RNDNRKT RSSNLSQ DNSSRIR**

Note: the rational design was not included in the library design because of compromising between codon degeneracy and library size.

**Z1166L4**

3’ g a c a a t g t g t a c 5’

| | | | | | | | | | | |

RSDNLSE QKANRTK RSDALSR NSSNRKV

**RRV S LKT RR R LTE**

**K R S K K S**

**N N R N N R**

**T T N T T N**

**I I I I I I**

n: 282 8 282 28 8 282 = ~34 million

(synonymous codons counted separately)

**>Library z1166L4**

CAATATTTCAAGCTATACCAAGCATACAATCAACTCCAAGCTTACCATGGCCGAGGAACGCCCCTTCCAGTGTCGCATCTGTATGCGCAATTTCAGCCGCAGCGACAACCKGANMGWGCACATTCGCACGCACACCGGCGAAAAGCCCTTTGCCTGTGACATTTGTGGGCGCAAGTTCGCGCAGANMGCCAACCKGANMAMGCATACCAAAATTCACACCGGATCTGAGCGCCCGTTTCAGTGCAGGATTTGCATGAGGAACTTCTCCCGGTCCGACGCCCKGANMCGCCATATCAGGACCCATACCGGGGAGAAACCTTTCGCGTGCGATATCTGCGGGAGGAAATTCGCCAACANMAGCAACCKGANMGWGCACACGAAGATCCATCAGAACAAGAAGCAACTAGTCAAAAGTGAACTAAGCTTTGCAAAGATGGATAAAGCGGAATTAATTCCCGAGCCT

Result: **RSDNLIV QNANRNT RSDALSR NSSNRTV**

z1166L only worked after Y1H optimisation (Fig. 3). Therefore the library worked even though it was undersampled.

**z1166R**

3’gg t g g t a g g t g a t 5’

| | | | | | | | | | | |

RSDTLSR RKDVRIY RSSHLST RSDNRTT

**K** **AA** **K** **K** **AA** **D** **K**  **R** **A** **K** **AT** **K**

**N**  **A**

**D**

n: 2 22 2 2 22 3 2 2 2 2 24 2 = 98,304

**>Library z1166R**

CAATATTTCAAGCTATACCAAGCATACAATCAACTCCAAGCTTACCATGGCCGAGGAACGCCCCTTCCAGTGTCGCATCTGTATGCGCAATTTCAGCARGAGCGMCRCCCTGAGCARGCACATTCGCACGCACACCGGCGAAAAGCCCTTTGCCTGTGACATTTGTGGGCGCAAGTTCGCGARGAAGGMCGYCCGCATCDACCATACCAAAATTCACACCGGATCTGAGCGCCCGTTTCAGTGCAGGATTTGCATGAGGAACTTCTCCARGAGCAGCCACCKGAGCRCCCATATCAGGACCCATACCGGGGAGAAACCTTTCGCGTGCGATATCTGCGGGAGGAAATTCGCCARGAGCGMCRMCCGCACCAMGCACACGAAGATCCATCAGAACAAGAAGCAACTAGTCAAAAGTGAACTAAGCTTTGCAAAGATGGATAAAGCGGAATTAATTCCCGAGCCT

Result: **RSDTLSR RKDARIN RSSHLST KSDNRTT**

z1166R improved after Y1H optimisation (Fig. 3). Therefore the library worked.

**4. Donor plasmid sequences:**

Homo sapiens (human) Build 36.3 (Current)The Human Genome NW_001838403

Homo sapiens chromosome 17 genomic contig, alternate assembly (based on HuRef SCAF_1103279188371)

771_target sites in green, 1166_target sites in blue (with silent “barcode” mutations highlighted in red).

Exons in grey. Mutation hot spots in red. XhoI sites in yellow.

Hotspots: ATG=133; CCG=152; CGC=175*; CGA=213; AAC=235; GGC=245*; CGG=248*; AGG=249*; CGT=273*; CGG=282

pUC57_truncated_p53_repair matrix (used in 293T, SF268)

TTGCGTGTGGAGTATTTGGATGACAGAAACACTTTTCGACATAGTGTGGTGGTGCCCTATGAGCCGCCTGAGGTCTGGTTTGCAACTGGGGTCTCTGGGAGGAGGGGTTAAGGGTGGTTGTCAGTGGCCCTCCGGGTGAGCAGTAGGGGGGCTTTCTCCTGCTGCTTATTTGACCTCCCTATAACCCCATGAGATGTGCAAAGTAAATGGGTTTAACTATTGCACAGTTGAAAAAACTGAAGCTTACAGAGGCTAAGGGCCTCCCCTGCTTGGCTGGCTGCTCGAGCTAGCGCAGTGGCTCATGCCTGTAATCCCAGCACTTTGGGAGGCCAAGGCAGGCGGATCACGAGGTTGGGAGATCGAGACCATCCTGGCTAACGGTGAAACCCCGTCTCTACTGAAAAATACAAAAAAAAATTAGCCGGGCGTGGTGCTGGGCACCTGTAGTCCCAGCTACTCGGGAGGCTGAGGAAGGAGAATGGCGTGAACCTGGGCGGTGGAGCTTGCAGTGAGCTGAGATCACGCCACTGCACTCCAGCCTGGGCGACAGAGCGAGATTCCATCTCAAAAAAAAAAAAAAAAGGCCTCCCCTGCTTGCCACAGGTCTCCCCAAGGCGCACTGGCCTCATCTTGGGCCTGTGTTATCTCCTAGGTTGGCTCTGACTGTACGacGatTcaTtacaactaTatgtgCaaTTCcTCCTGCATGGGCGGCATGAACCGGAGGCCCATCCTCACCATCATCACACTGGAAGACTCCAGGTCAGGAGCCACTTGCCACCCTGCACACTGGCCTGCTGTGCCCCAGCCTCTGCTTGCCTCTGACCCCTGGGCCCACCTCTTACCGATTTCTTCCATACTACTACCCATCCACCTCTCATCACATCCCCGGCGGGGAATCTCCTTACTGCTCCCACTCAGTTTTCTTTTCTCTGGCTTTGGGACCTCTTAACCTGTGGCTTCTCCTCCACCTACCTGGAGCTGGAGCTTAGGCTCCAGAAAGGACAAGGGTGGTTGGGAGTAGATGGAGCCTGGTTTTTTAAATGGGACAGGTAGGACCTGATTTCCTTACTGCCTCTTGCTTCTCTTTTCCTATCCTGAGTAGTGGTAATCTACTGGGACGGAACAGCTTTGAGGTGCGTGTTTGTGCCTGTCCTGGGAGAGACCGGCGCACAGAGGAAGAGAATCTCCGCAAGAAAGGGGAGCCTCACCACGAGCTGCCCCCAGGGAGCACTAAGCGAGGTAAGCAAGCAGGACAAGAAGCGGTGGAGGAGACCAAGGGTGCAGTTATGCCTCAGATTCACTTTTATCACCTTTCCTTGCCTCTT**TCCTAGCACTGCCCAACAAC**

pUC57_p53_repair matrix_771(used in 293T)

AAGAAAAGCTCCTGAGGTGTAGACGCCAACTCTCTCTAGCTCGCTAGTGGGTTGCAGGAGGTGCTTACACATGTTTGTTTCTTTGCTGCCGTCTTCCAGTTGCTTTATCTGTTCACTTGTGCCCTGACTTTCAACTCTGTCTCCTTCCTCTTCCTACAGTACTCCCCTGCCCTCAACAAGATGTTTTGCCAACTGGCCAAGACCTGCCCTGTGCAGCTGTGGGTTGATTCCACACCCCCGCCCGGCACCCGCGTCCGCGCCATGGCCATCTACAAGCAGTCACAGCACATGACGGAGGTTGTGAGGCGCTGCCCCCACCATGAGCGCTGCTCAGATAGCGATGGTGAGCAGCTGGGGCTGGAGAGACGACAGGGCTGGTTGCCCAGGGTCCCCAGGCCTCTGATTCCTCACTGATTGCTCTTAGGTCTGGCCCCTCCTCAGCATCTTATCCGAGTGGAAGGAAATTTGCGTGTGGAGTATTTGGATGACAGAAACACTTTTCGACATAGTGTGGTGGTGCCCTATGAGCCGCCTGAGGTCTGGTTTGCAACTGGGGTCTCTGGGAGGAGGGGTTAAGGGTGGTTGTCAGTGGCCCTCCGGGTGAGCAGTAGGGGGGCTTTCTCCTGCTGCTTATTTGACCTCCCTATAACCCCATGAGATGTGCAAAGTAAATGGGTTTAACTATTGCACAGTTGAAAAAACTGAAGCTTACAGAGGCTAAGGGCCTccGTtgTttggTtgggcgcaCAggAtcTACCCTGTAATCCCAGCACTTTGGGAGGCCAAGGCAGGCGGATCACGAGGTTGGGAGATCGAGACCATCCTGGCTAACGGTGAAACCCCGTCTCTACTGAAAAATACAAAAAAAAATTAGCCGGGCGTGGTGCTGGGCACCTGTAGTCCCAGCTACTCGGGAGGCTGAGGAAGGAGAATGGCGTGAACCTGGGCGGTGGAGCTTGCAGTGAGCTGAGATCACGCCACTGCACTCCAGCCTGGGCGACAGAGCGAGATTCCATCTCAAAAAAAAAAAAAAAAGGCCTCCCCTGCTTGCCACAGGTCTCCCCAAGGCGCACTGGCCTCATCTTGGGCCTGTGTTATCTCCTAGGTTGGCTCTGACTGTACCACCATCCACTACAACTACATGTGTAACAGTTCCTGCATGGGCGGCATGAACCGGAGGCCCATCCTCACCATCATCACACTGGAAGACTCCAGGTCAGGAGCCACTTGCCACCCTGCACACTGGCCTGCTGTGCCCCAGCCTCTGCTTGCCTCTGACCCCTGGGCCCACCTCTTACCGATTTCTTCCATACTACTACCCATCCACCTCTCATCACATCCCCGGCGGGGAATCTCCTTACTGCTCCCACTCAGTTTTCTTTTCTCTGGCTTTGGGACCTCTTAACCTGTGGCTTCTCCTCCACCTACCTGGAGCTGGAGCTTAGGCTCCAGAAAGGACAAGGGTGGTTGGGAGTAGATGGAGCCTGGTTTTTTAAATGGGACAGGTAGGACCTGATTTCCTTACTGCCTCTTGCTTCTCTTTTCCTATCCTGAGTAGTGGTAATCTACTGGGACGGAACAGCTTTGAGGTGCGTGTTTGTGCCTGTCCTGGGAGAGACCGGCGCACAGAGGAAGAGAATCTCCGCAAGAAAGGGGAGCCTCACCACGAGCTGCCCCCAGGGAGCACTAAGCGAGGTAAGCAAGCAGGACAAGAAGCGGTGGAGGAGACCAAGGGTGCAGTTATGCCTCAGATTCACTTTTATCACCTTTCCTTGCCTCTT

**5. Yeast and genomic PCRs**

Yeast colony PCR:

**pADH1_HA_Frw:** CAATATTTCAAGCTATACCAAGCATACAATCAACTCCAAGCTTACCATGGCCGAGGAACGCCCCTTCCAGTGTCGCATC

**GAL4_HA_Rev:** AGGCTCGGGAATTAATTCCGCTTTATCCATCTTTGCAAAGCTTAGTTCACTTTTGACTAGTTGCTTCTTGTTCTGATGGATC

Amplification conditions: 98 °C for 8 min, then 25 cycles of 95 °C for 30 sec, 55°C for 30 sec and 72 °C for 1 min, followed by extension at 72 °C for 3 min.

Nested PCR to introduce T7 promoter:

**T7 Kozak Fwd_phage:** TCGAGTAATACGACTCACTATAGGGAGAAACACCATAGATTGCCATGGCCGAGGAACGCCCCTTC

**ZFP_LVKSEL_Rev fusion:** CAGTTCACTTTTGACTAG

Amplification conditions: 95 °C for 3 min, then 25 cycles of 95 °C for 30 sec, 55°C for 30 sec and 72 °C for 1 min, followed by extension at 72 °C for 3 min.

FokI PCR:

**FokI_LVKSEL_Fwd_fusion:** CTAGTCAAAAGTGAACTG

**2xFLAG_REV:** CTCACTACTTGTCATCGTCATCCTTGTAGTCACCGTCATGGTCTTTGTAGTCTGCGGCAAAGTTTATCTCGCCGTTATTAAATTTCCG

Amplification conditions: 95 °C for 3 min, then 30 cycles of 95 °C for 30 sec, 58 °C for 30 sec and 72 °C for 1 min, followed by extension at 72 °C for 3 min.

Fusion PCR of ZFP and FokI:

**T7 Kozak Fwd_phage:**

TCGAGTAATACGACTCACTATAGGGAGAAACACCATAGATTGCCATGGCCGAGGAACGCCCCTTC

**2xFLAG_REV:**

CTCACTACTTGTCATCGTCATCCTTGTAGTCACCGTCATGGTCTTTGTAGTCTGCGGCAAAGTTTATCTCGCCGTTATTAAATTTCCG

Amplification conditions: 95 °C for 5 min, then 25 cycles of 95 °C for 20 sec, 55°C for 20 sec and 72 °C for 1 min, followed by extension at 72 °C for 3 min.

Genomic Integration of p53 donor sequence

Genomic PCR (HEK293T, SF268, and BT-549 cell lines)

**p53_diagnostic_fwd:** GTCTGGCCCCTCCTCAGCATCTTATCCGAG

**p53_diagnostic_external_reverse:** CAAGACTTAGTACCTGAAGGGTG

Amplification conditions: 95 °C for 3 min, then 25 cycles of 95 °C for 30 sec, 56°C for 30 sec and 68 °C for 1 min, followed by extension at 68 °C for 3 min. The expected amplicon length is 1448 bp.

Genomic PCR (K562)

**771F_wt:** CCCCTGCTTGGCTGGGCGCAGTGGCTCATG

**Diagnostic_external_R:** GGTTTCTTCTTTGGCTGGGGAGAGGAGC

Amplification conditions: 95 °C for 5 min, then 10 cycles of 95 °C for 30 sec (decrease 0.5 °C every cycle), 68 °C for 1 min 30 sec then 10 cycles of 95 °C for 30 sec, 58°C for 30 sec and 72 °C for 1 min 20 sec, followed by extension at 72 °C for 5 min. The expected amplicon length is 1105 bp (wt) and 1117 bp (modified).

Discrimination between the wild type and the integrated modified p53 sequence

(Nested “barcode” PCR)

z1166 site in K562 cells:

**1166_Xho_I_diag_fwd_alternate (K562):** ACCACCATCCACTACAACTGCTCGAGC

**Diagnostic_external_R:** GGTTTCTTCTTTGGCTGGGGAGAGGAGC

z1166 site in HEK293T and SF268 cells:

**1166F_repaired_short1**: GACGATTCATTACAACTATATGTGCAATTC

**p53_diagnostic_external_reverse:** CAAGACTTAGTACCTGAAGGGTG

z771 site in HEK293T cells:

**771F_repair:** CCGTTGTTTGGTTGGGCGCACAGGATCTAC

**p53_diagnostic_external_reverse:** CAAGACTTAGTACCTGAAGGGTG

Amplification conditions: 95 °C for 3 min, then 35 (wt control: 22) cycles of 95 °C for 30 sec, 60 °C for 25 sec and 72 °C for 1 min, followed by extension at 72 °C for 3 min.

**6. Solexa deep sequencing protocols**

**6.1 Step 1.** **Genomic PCR** as above

**6.2 Step 2.** S**equencing barcode PCR** (3bp-xxx)

Forward oligos: (MmeI site in blue, 3 base solexa barcode in yellow).

Note that MmeI will cut at:

5'-TCCRAC(N_20_)-3'

3'-AGGYTG(N_18_)-5'

i.e. at TA below:

new1166_solexa_F_ATC

TCTTGGtccgacGGCGCACTGGCCTCATCTTAGGCATCTGTTATCTCCTAGGTTGGCTCTG

new1166_solexa_F_TTG

TCTTGGtccgacGGCGCACTGGCCTCATCTTAGGCTTGTGTTATCTCCTAGGTTGGCTCTG

new1166_solexa_F_CCT

TCTTGGtccgacGGCGCACTGGCCTCATCTTAGGCCCTTGTTATCTCCTAGGTTGGCTCTG

new1166_solexa_F_GGA

TCTTGGtccgacGGCGCACTGGCCTCATCTTAGGCGGATGTTATCTCCTAGGTTGGCTCTG

new1166_solexa_F_CAG

TCTTGGtccgacGGCGCACTGGCCTCATCTTAGGCCAGTGTTATCTCCTAGGTTGGCTCTG

new1166_solexa_F_CGC

TCTTGGtccgacGGCGCACTGGCCTCATCTTAGGCCGCTGTTATCTCCTAGGTTGGCTCTG

new1166_solexa_F_GCC

TCTTGGtccgacGGCGCACTGGCCTCATCTTAGGCGCCTGTTATCTCCTAGGTTGGCTCTG

new1166_solexa_F_GTT

TCTTGGtccgacGGCGCACTGGCCTCATCTTAGGCGTTTGTTATCTCCTAGGTTGGCTCTG

Reverse:

1166_solexa_R: GCACAGCAGGCCAGTGTG

Amplification conditions: 95 °C for 3 min, then 20 cycles of 95 °C for 20 sec, 62 °C for 20 sec and 68 °C for 30 secs.

**6.2.1 Protocol with MmeI digestion**

For short 31bp Solexa reads only:

Pool 8 different barcode solexa PCRs per solexa lane

Minelute to ~75ng/ul = 1.5ug (cut all with MmeI)

0.5 ul bsa

5 ul NEB4

20 ul DNA (1.5ug)

1ul SAM 50X (dilute 32mM stock: 1 in 12.8ul H2O for 50X)

0.75 ul MmeI

22.75 H2O

----

50 ul -> 37˚C, 15 min -> minelute immediately, elute in 34ul EB.

Solexa run: single-end read using manufacturer's protocol on Genome Analyser II machine.

**6.2.2 Protocol without MmeI digestion**

PCR above, followed by second PCR: Solexa1_F/1166_solexa_R

(Solexa1_F: CTGGCCTCATCTTAG):

95 °C for 3 min, then 10 cycles of 95 °C for 20 sec, 50 °C for 10 sec and 68 °C for 10 secs. End with 68 °C for 5 min and 18˚C, hold. Use 50 ng template PCR per 50ul PCR.

**Notes**:

To avoid PCR cross-over artefacts, barcoded samples should be amplified and prepared for solexa separately, mixing should be done just prior to loading onto flow cells. PCR cycles should be kept to a minimum. If possible, include barcodes and sequencing adapters in 1 PCR step.

**Resulting PCR templates for deep sequencing:**

(ZFP sites; sequencing barcodes: XXX; mutant donor insert barcodes: X; data filters underlined)

>184bp-wt

CTGGCCTCATCTTAGGCxxxTGTTATCTCCTAGGTTGGCTCTGACTGTAC**CACCATCCACTACAACTACATGTGTAACAG**TTCCTGCATGGGCGGCATGAACCGGAGGCCCATCCTCACCATCATCACACTGGAAGACTCCAGGTCAGGAGCCACTTGCCACCCTGCACACTGGCCTGCTGTGC

>184bp-mut

CTGGCCTCATCTTAGGCxxxTGTTATCTCCTAGGTTGGCTCTGACTGTACGACGATTCATTACAACTATATGTGCAATTCCTCCTGCATGGGCGGCATGAACCGGAGGCCCATCCTCACCATCATCACACTGGAAGACTCCAGGTCAGGAGCCACTTGCCACCCTGCACACTGGCCTGCTGTGC

These PCRs were given to the CRG deep sequencing facility for Solexa adapter ligation and 104bp paired-end reads. Note use of 50% phiX spiking and GA IIx machine for good reads (not HiSeq machine).

**6.3 Step 3. Data processing**

Extract sequence if it contains a defined prefix and suffix (perl script):

**Prefix Suffix**

NHEJ 1st filter (wt)

GGCXXXTGT TTCCTGCAT

NHEJ 2nd filter (30bp around ZFN site)

CTGTAC TTCCTG

Barcode filter (gene repair)

GGCXXXTGT GACGATTCAT

**7. Measurement of double-stranded breaks in ZFN-treated cells**

**Protocol**

HEK293T cells were grown as described above and transfected with a total of 200 ng ZFN expression vectors. As controls 200ng of I-SceI expression vector and 200ng of pUC19 were used. As a positive control for the induction of double stranded breaks, cells were treated with etoposide (1mM) for 5 hrs. After 48 hrs cells were harvested and subjected to staining with antibodies against γH2A.X using the H2A.X Phosphorylation Assay Kit for Flow Cytometry (Upstate 17-344) following the manufacturer’s protocol. Stained cells were analyzed using a Guava Easycyte system.

**8. DNA and protein sequences of zinc finger-FokI constructs**

Note: for chromosomal assays, obligate heterodimer versions of the FokI were introduced, using the mutations from: Miller et al., Nat. Biotech 2007, PMID 17603475.

**Key**

ATG=start codon

PKKKRKV=SV40 nuclear localisation signal

ZFP coding region (alpha helices underlined from position –1 to position +6)

ACTAGT SpeI

FokI coding region

TAA=stop codon

>z771L_A6.6_NLS

ATGGCCCCCAAAAAGAAGCGCAAGGTCGCCGAGGAACGCCCCTTCCAGTGTCGCATCTGTATGCGCAATTTCAGCCGCAACTCCAGCCTGACGAACCACATTCGCACGCACACCGGCGAAAAGCCCTTTGCCTGTGACATTTGTGGGCGCAAGTTCGCGGCCACCAACTCCCTCATAGAACATACCAAAATTCACACCGGATCCGAGCGCCCGTTTCAGTGCAGGATTTGCATGAGGAACTTCTCCCGCTCCTGCCACCTGAAGACCCATATCAGGACCCATACCGGGGAGAAACCTTTCGCGTGCGATATCTGCGGGAGGAAATTCGCCCGCAGCGACAACCTGAAGACACACACGAAGATCCATCAGAACAAGAAGCAACTAGTCAAAAGTGAACTGGAGGAGAAGAAATCTGAACTTCGTCATAAATTGAAATATGTGCCTCATGAATATATTGAATTAATTGAAATTGCCAGAAATTCCACTCAGGATAGAATTCTTGAAATGAAGGTAATGGAATTTTTTATGAAAGTTTATGGATATAGAGGTAAACATTTGGGTGGATCAAGGAAACCGGACGGAGCAATTTATACTGTCGGATCTCCTATTGATTACGGTGTGATCGTGGATACTAAAGCTTATAGCGGAGGTTATAATCTGCCAATTGGCCAAGCAGATGAAATGCAACGATATGTCGAAGAAAATCAAACACGAAACAAACATATCAACCCTAATGAATGGTGGAAAGTCTATCCATCTTCTGTAACGGAATTTAAGTTTTTATTTGTGAGTGGTCACTTTAAAGGAAACTACAAAGCTCAGCTTACACGATTAAATCATATCACTAATTGTAATGGAGCTGTTCTTAGTGTAGAAGAGCTTTTAATTGGTGGAGAAATGATTAAAGCCGGCACATTAACCTTAGAGGAAGTGAGACGGAAATTTAATAACGGCGAGATAAACTTTTAA

>z771L_A6.6

MAPKKKRKVAEERP

FQCRICMRNFSRNSSLTNHIRTHTGEKP

FACDICGRKFAATNSLIEHTKIHTGSERP

FQCRICMRNFSRSCHLKTHIRTHTGEKP

FACDICGRKFARSDNLKTHTKIHQNKKQ

LVKSELEEKKSELRHKLKYVPHEYIELIEIARNSTQDRILEMKVMEFFMKVYGYRGKHLGGSRKPDGAIYTVGSPIDYGVIVDTKAYSGGYNLPIGQADEMQRYVEENQTRNKHINPNEWWKVYPSSVTEFKFLFVSGHFKGNYKAQLTRLNHITNCNGAVLSVEELLIGGEMIKAGTLTLEEVRRKFNNGEINF*

>z771Rmod_NLS_remut

ATGGCCCCCAAAAAGAAGCGCAAGGTCGCCGAGCGCCCCTTCCAGTGTCGCATCTGTATGCGCAATTTCAGCCGCAGCAGCCACCTGAGCCGCCACATTCGCACGCACACCGGCGAAAAGCCCTTTGCCTGTGACATTTGTGGGCGCAAGTTCGCGCGCAACGACAACCGCAAGACCCATACCAAAATTCACACCGGCGGACAGCGGCCGTTTCAGTGCAGGATTTGCATGAGGAACTTCTCCCGCAGCAGCAACCTGAGCCAGCATATCAGGACCCATACCGGGGAGAAACCTTTCGCGTGCGATATCTGCGGGAGGAAATTCGCCGACAACAGCAGCCGCATCCGCCACACGAAGATCCATCAGAACAAGAAGCAACTAGTCAAAAGTGAACTGGAGGAGAAGAAATCTGAACTTCGTCATAAATTGAAATATGTGCCTCATGAATATATTGAATTAATTGAAATTGCCAGAAATTCCACTCAGGATAGAATTCTTGAAATGAAGGTAATGGAATTTTTTATGAAAGTTTATGGATATAGAGGTAAACATTTGGGTGGATCAAGGAAACCGGACGGAGCAATTTATACTGTCGGATCTCCTATTGATTACGGTGTGATCGTGGATACTAAAGCTTATAGCGGAGGTTATAATCTGCCAATTGGCCAAGCAGATGAAATGCAACGATATGTCGAAGAAAATCAAACACGAAACAAACATATCAACCCTAATGAATGGTGGAAAGTCTATCCATCTTCTGTAACGGAATTTAAGTTTTTATTTGTGAGTGGTCACTTTAAAGGAAACTACAAAGCTCAGCTTACACGATTAAATCATATCACTAATTGTAATGGAGCTGTTCTTAGTGTAGAAGAGCTTTTAATTGGTGGAGAAATGATTAAAGCCGGCACATTAACCTTAGAGGAAGTGAGACGGAAATTTAATAACGGCGAGATAAACTTTTAA

>z771Rmod_NLS_remut

MAPKKKRKVAERP

FQCRICMRNFSRSSHLSRHIRTHTGEKP

FACDICGRKFARNDNRKTHTKIHTGGQRP

FQCRICMRNFSRSSNLSQHIRTHTGEKP

FACDICGRKFADNSSRIRHTKIHQNKKQ

LVKSELEEKKSELRHKLKYVPHEYIELIEIARNSTQDRILEMKVMEFFMKVYGYRGKHLGGSRKPDGAIYTVGSPIDYGVIVDTKAYSGGYNLPIGQADEMQRYVEENQTRNKHINPNEWWKVYPSSVTEFKFLFVSGHFKGNYKAQLTRLNHITNCNGAVLSVEELLIGGEMIKAGTLTLEEVRRKFNNGEINF*

>z1166L_C1.3a_NLS

ATGGCCCCCAAAAAGAAGCGCAAGGTCGCCGAGGAACGCCCCTTCCAGTGTCGCATCTGTATGCGCAATTTCAGCCGCAGCGACAACCTGATAGTGCACATTCGCACGCACACCGGCGAAAAGCCCTTTGCCTGTGACATTTGTGGGCGCAAGTTCGCGCAGAACGCCAACCGGAACACGCATACCAAAATTCACACCGGATCTGAGCGCCCGTTTCAGTGCAGGATTTGCATGAGGAACTTCTCCCGGTCCGACGCCCTGAGCCGCCATATCAGGACCCATACCGGGGAGAAACCTTTCGCGTGCGATATCTGCGGGAGGAAATTCGCCAACAGCAGCAACCGGACAGTGCACACGAAGATCCATCAGAACAAGAAGCAACTAGTCAAAAGTGAACTGGAGGAGAAGAAATCTGAACTTCGTCATAAATTGAAATATGTGCCTCATGAATATATTGAATTAATTGAAATTGCCAGAAATTCCACTCAGGATAGAATTCTTGAAATGAAGGTAATGGAATTTTTTATGAAAGTTTATGGATATAGAGGTAAACATTTGGGTGGATCAAGGAAACCGGACGGAGCAATTTATACTGTCGGATCTCCTATTGATTACGGTGTGATCGTGGATACTAAAGCTTATAGCGGAGGTTATAATCTGCCAATTGGCCAAGCAGATGAAATGCAACGATATGTCGAAGAAAATCAAACACGAAACAAACATATCAACCCTAATGAATGGTGGAAAGTCTATCCATCTTCTGTAACGGAATTTAAGTTTTTATTTGTGAGTGGTCACTTTAAAGGAAACTACAAAGCTCAGCTTACACGATTAAATCATATCACTAATTGTAATGGAGCTGTTCTTAGTGTAGAAGAGCTTTTAATTGGTGGAGAAATGATTAAAGCCGGCACATTAACCTTAGAGGAAGTGAGACGGAAATTTAATAACGGCGAGATAAACTTTTAA

>z1166L_C1.3a_NLS

MAPKKKRKVAEERP

FQCRICMRNFSRSDNLIVHIRTHTGEKP

FACDICGRKFAQNANRNTHTKIHTGSERP

FQCRICMRNFSRSDALSRHIRTHTGEKP

FACDICGRKFANSSNRTVHTKIHQNKKQ

LVKSELEEKKSELRHKLKYVPHEYIELIEIARNSTQDRILEMKVMEFFMKVYGYRGKHLGGSRKPDGAIYTVGSPIDYGVIVDTKAYSGGYNLPIGQADEMQRYVEENQTRNKHINPNEWWKVYPSSVTEFKFLFVSGHFKGNYKAQLTRLNHITNCNGAVLSVEELLIGGEMIKAGTLTLEEVRRKFNNGEINF*

>z1166R_M9_NLS

ATGGCCCCCAAAAAGAAGCGCAAGGTCGCCGAGGAACGCCCCTTCCAGTGTCGCATCTGTATGCGCAATTTCAGCAGGAGCGACACCCTGAGCAGGCACATTCGCACGCACACCGGCGAAAAGCCCTTTGCCTGTGACATTTGTGGGCGCAAGTTCGCGAGGAAGGACGCCCGCATCAACCATACCAAAATTCACACCGGATCTGAGCGCCCGTTTCAGTGCAGGATTTGCATGAGGAACTTCTCCAGGAGCAGCCACCTGAGCACCCATATCAGGACCCATACCGGGGAGAAACCTTTCGCGTGCGATATCTGCGGGAGGAAATTCGCCAAGAGCGACAACCGCACCACGCACACGAAGATCCATCAGAACAAGAAGCAACTAGTCAAAAGTGAACTGGAGGAGAAGAAATCTGAACTTCGTCATAAATTGAAATATGTGCCTCATGAATATATTGAATTAATTGAAATTGCCAGAAATTCCACTCAGGATAGAATTCTTGAAATGAAGGTAATGGAATTTTTTATGAAAGTTTATGGATATAGAGGTAAACATTTGGGTGGATCAAGGAAACCGGACGGAGCAATTTATACTGTCGGATCTCCTATTGATTACGGTGTGATCGTGGATACTAAAGCTTATAGCGGAGGTTATAATCTGCCAATTGGCCAAGCAGATGAAATGCAACGATATGTCGAAGAAAATCAAACACGAAACAAACATATCAACCCTAATGAATGGTGGAAAGTCTATCCATCTTCTGTAACGGAATTTAAGTTTTTATTTGTGAGTGGTCACTTTAAAGGAAACTACAAAGCTCAGCTTACACGATTAAATCATATCACTAATTGTAATGGAGCTGTTCTTAGTGTAGAAGAGCTTTTAATTGGTGGAGAAATGATTAAAGCCGGCACATTAACCTTAGAGGAAGTGAGACGGAAATTTAATAACGGCGAGATAAACTTTTAA

>z1166R_M9_NLS

MAPKKKRKVAEERP

FQCRICMRNFSRSDTLSRHIRTHTGEKP

FACDICGRKFARKDARINHTKIHTGSERP

FQCRICMRNFSRSSHLSTHIRTHTGEKP

FACDICGRKFAKSDNRTTHTKIHQNKKQ

LVKSELEEKKSELRHKLKYVPHEYIELIEIARNSTQDRILEMKVMEFFMKVYGYRGKHLGGSRKPDGAIYTVGSPIDYGVIVDTKAYSGGYNLPIGQADEMQRYVEENQTRNKHINPNEWWKVYPSSVTEFKFLFVSGHFKGNYKAQLTRLNHITNCNGAVLSVEELLIGGEMIKAGTLTLEEVRRKFNNGEINF*

Obligate heterodimer FokIs:

From Miller et al., Nat. Biotech 2007, PMID 17603475

>negatively -charged monomer

CTAGTCAAAAGTGAACTGGAGGAGAAGAAATCTGAACTTCGTCATAAATTGAAATATGTGCCTCATGAATATATTGAATTAATTGAAATTGCCAGAAATTCCACTCAGGATAGAATTCTTGAAATGAAGGTAATGGAATTTTTTATGAAAGTTTATGGATATAGAGGTAAACATTTGGGTGGATCAAGGAAACCGGACGGAGCAATTTATACTGTCGGATCTCCTATTGATTACGGTGTGATCGTGGATACTAAAGCTTATAGCGGAGGTTATAATCTGCCAATTGGCCAAGCAGATGAAATGgAgCGATATGTCGAAGAAAATCAAACACGAAACAAACATcTCAACCCTAATGAATGGTGGAAAGTCTATCCATCTTCTGTAACGGAATTTAAGTTTTTATTTGTGAGTGGTCACTTTAAAGGAAACTACAAAGCTCAGCTTACACGATTAAATCATATCACTAATTGTAATGGAGCTGTTCTTAGTGTAGAAGAGCTTTTAATTGGTGGAGAAATGATTAAAGCCGGCACATTAACCTTAGAGGAAGTGAGACGGAAATTTAATAACGGCGAGATAAACTTTTAA

>negative

LVKSELEEKKSELRHKLKYVPHEYIELIEIARNSTQDRILEMKVMEFFMK

VYGYRGKHLGGSRKPDGAIYTVGSPIDYGVIVDTKAYSGGYNLPIGQADE

MERYVEENQTRNKHLNPNEWWKVYPSSVTEFKFLFVSGHFKGNYKAQLTR

LNHITNCNGAVLSVEELLIGGEMIKAGTLTLEEVRRKFNNGEINF*

>positively-charged monomer

CTAGTCAAAAGTGAACTGGAGGAGAAGAAATCTGAACTTCGTCATAAATTGAAATATGTGCCTCATGAATATATTGAATTAATTGAAATTGCCAGAAATTCCACTCAGGATAGAATTCTTGAAATGAAGGTAATGGAATTTTTTATGAAAGTTTATGGATATAGAGGTAAACATTTGGGTGGATCAAGGAAACCGGACGGAGCAATTTATACTGTCGGATCTCCTATTGATTACGGTGTGATCGTGGATACTAAAGCTTATAGCGGAGGTTATAATCTGCCAATTGGCCAAGCAGATGAAATGCAACGATATGTCaAAGAAAATCAAACACGAAACAAACATATCAACCCTAATGAATGGTGGAAAGTCTATCCATCTTCTGTAACGGAATTTAAGTTTTTATTTGTGAGTGGTCACTTTAAAGGAAACTACAAAGCTCAGCTTACACGATTAAATCATAagACTAATTGTAATGGAGCTGTTCTTAGTGTAGAAGAGCTTTTAATTGGTGGAGAAATGATTAAAGCCGGCACATTAACCTTAGAGGAAGTGAGACGGAAATTTAATAACGGCGAGATAAACTTTTAA

>positive

LVKSELEEKKSELRHKLKYVPHEYIELIEIARNSTQDRILEMKVMEFFMK

VYGYRGKHLGGSRKPDGAIYTVGSPIDYGVIVDTKAYSGGYNLPIGQADE

MQRYVKENQTRNKHINPNEWWKVYPSSVTEFKFLFVSGHFKGNYKAQLTR

LNHKTNCNGAVLSVEELLIGGEMIKAGTLTLEEVRRKFNNGEINF*
